# NMDA receptor autoantibodies primarily impair the extrasynaptic compartment

**DOI:** 10.1093/brain/awae163

**Published:** 2024-05-17

**Authors:** Zoe Jamet, Camille Mergaux, Morgane Meras, Delphine Bouchet, Frédéric Villega, Jakob Kreye, Harald Prüss, Laurent Groc

**Affiliations:** Interdisciplinary Institute for Neuroscience, IINS, UMR 5297, University of Bordeaux, CNRS, F-33000 Bordeaux, France; Interdisciplinary Institute for Neuroscience, IINS, UMR 5297, University of Bordeaux, CNRS, F-33000 Bordeaux, France; Interdisciplinary Institute for Neuroscience, IINS, UMR 5297, University of Bordeaux, CNRS, F-33000 Bordeaux, France; Interdisciplinary Institute for Neuroscience, IINS, UMR 5297, University of Bordeaux, CNRS, F-33000 Bordeaux, France; Interdisciplinary Institute for Neuroscience, IINS, UMR 5297, University of Bordeaux, CNRS, F-33000 Bordeaux, France; Department of Pediatric Neurology, CIC-0005, University Children's Hospital of Bordeaux, F-33000 Bordeaux, France; German Center for Neurodegenerative Diseases (DZNE) Berlin, 10117 Berlin, Germany; Department of Neurology and Experimental Neurology, Charité-Universitätsmedizin Berlin, Corporate Member of Freie Universität Berlin, Humboldt-Universität Berlin, 10117 Berlin, Germany; German Center for Neurodegenerative Diseases (DZNE) Berlin, 10117 Berlin, Germany; Department of Neurology and Experimental Neurology, Charité-Universitätsmedizin Berlin, Corporate Member of Freie Universität Berlin, Humboldt-Universität Berlin, 10117 Berlin, Germany; Interdisciplinary Institute for Neuroscience, IINS, UMR 5297, University of Bordeaux, CNRS, F-33000 Bordeaux, France

**Keywords:** autoantibody, encephalitis, extrasynaptic NMDA receptor, membrane proteins, interactome

## Abstract

Autoantibodies directed against the *N*-methyl-D-aspartate receptor (NMDAR-Ab) are pathogenic immunoglobulins detected in patients suffering from NMDAR encephalitis. NMDAR-Ab alter the receptor membrane trafficking, synaptic transmission and neuronal network properties, leading to neurological and psychiatric symptoms in patients. Patients often have very little neuronal damage but rapid and massive (treatment-responsive) brain dysfunctions related to an unknown early mechanism of NMDAR-Ab. Our understanding of this early molecular cascade remains surprisingly fragmented.

Here, we used a combination of single molecule-based imaging of membrane proteins to unveil the spatiotemporal action of NMDAR-Ab on live hippocampal neurons.

We first demonstrate that different clones of NMDAR-Ab primarily affect extrasynaptic (and not synaptic) NMDARs. In the first minutes, NMDAR-Ab increase extrasynaptic NMDAR membrane dynamics, declustering its surface interactome. NMDAR-Ab also rapidly reshuffle all membrane proteins located in the extrasynaptic compartment. Consistent with this alteration of multiple proteins, effects of NMDAR-Ab were not mediated through the sole interaction between the NMDAR and EphB2 receptor. In the long term, NMDAR-Ab reduce the NMDAR synaptic pool by slowing down receptor membrane dynamics in a cross-linking-independent manner. Remarkably, exposing only extrasynaptic NMDARs to NMDAR-Ab was sufficient to produce their full-blown effect on synaptic receptors.

Collectively, we demonstrate that NMDAR-Ab initially impair extrasynaptic proteins, then the synaptic ones. These data thus shed new and unsuspected light on the mode of action of NMDAR-Ab and, probably, our understanding of (extra)synaptopathies.

## Teaser

Jamet *et al.* explore the effects of autoantibodies against the NMDA receptor on rodent hippocampal neurons. They describe the molecular cascade triggered by the autoantibodies in the first minutes to hours of exposure, and show that the autoantibodies initially target the extrasynaptic compartment.

**See Zhao *et al*. (https://doi.org/10.1093/brain/awae236) for a scientific commentary on this article.**

## Introduction

Over the past decades, the identification of autoimmune neurological and psychiatric disorders with patients expressing autoantibodies directed against membrane proteins has flourished.^1^ The most prominent autoantibody-mediated brain disorder is *N*-methyl-D-aspartate receptor (NMDAR) encephalitis, in which patients develop antibodies directed against the extracellular N-terminal domain of the obligatory GluN1-NMDAR subunit of the NMDAR (NMDAR-Ab).^2-5^ The patients present with a spectrum of severe neurological features (e.g. seizures) and psychiatric symptoms (e.g. psychosis) that could result in a persistent coma, illustrating the complexity of the neuropsychiatric features induced by NMDAR-Ab.^6^ Mechanistically, it has been proposed that NMDAR-Ab binding destabilizes the receptor through a weakening of the interaction with the EphB2 receptor (EphB2R).^7,8^ Over time, this destabilization increases the surface diffusion of synaptic NMDARs and promotes their displacement to the extrasynaptic compartment.^7,9^ Then, the bivalency of autoantibodies would cross-link receptors and favour their internalization, decreasing NMDAR plasma membrane content and global signalling.^5,10^

Although there is a full consensus that NMDAR-Ab trigger NMDAR hypofunction at hippocampal and cortical neurons,^11^ our understanding of the molecular cascade underpinning this phenotype remains fragmented, with different scenarios being equally supported by the existing data.^12^ For instance, NMDAR-Ab alter the surface trafficking^7^ and nano-organization^9^ of both synaptic and extrasynaptic NMDARs, leaving open the question of whether synaptic and extrasynaptic receptor compartments are concomitantly or sequentially impacted. On the one hand, the high density of NMDARs within the postsynaptic density^13^ would favour NMDAR-Ab action on the synaptic pool. On the other hand, the high density and crowding of transmembrane proteins located within the synaptic cleft (∼20 nm wide) would restrain NMDAR-Ab (∼12 nm) penetration within synapses and favour their binding to extrasynaptic NMDARs. Furthermore, NMDARs interact with a dozen transmembrane receptors and ion channels located within synapses or, for the majority, outside synapses.^14-16^ We previously showed that NMDAR-Ab weaken the interaction between the NMDAR and EphB2R,^7^ which regulates the NMDAR synaptic pool.^17-19^ However, NMDARs and EphB2Rs are present in both synaptic and extrasynaptic membrane compartments, complicating the interpretation of the corrupted interaction by NMDAR-Ab. In addition, these autoantibodies alter the membrane clustering and surface dynamics of the dopamine receptor, which is an NMDAR protein–protein interactor located solely in the extrasynaptic compartment.^20-22^ Whether NMDAR-Ab impact other partners of the NMDAR in a similar manner remains an open question. Finally, NMDAR-Ab bivalency would cross-link NMDARs and trigger their internalization, a process that massively reduces the receptor membrane diffusion. Yet, it has been reported that NMDAR-Ab can also increase the surface diffusion of some NMDARs exiting synapses and exploring the extrasynaptic membrane compartment,^7^ inconsistent with such a receptor cross-linking process. Given that other autoantibodies directed against different neurotransmitter receptors can alter their membrane signalling through a cross-linking-independent process,^23-25^ it remains unclear whether the action of NMDAR-Ab on NMDAR surface organization relies solely on autoantibody-induced cross-linking. Therefore, our lack of understanding of the spatiotemporal action of NMDAR-Ab on its target and other related membrane proteins strongly hampers our capacity to draw a comprehensive scenario of the molecular cascade triggered by autoantibodies. To address this question directly, we used a combination of single molecule-based imaging of various membrane proteins to unveil the effects of NMDAR-Ab on live hippocampal neurons. We took advantage of the recently developed patient-derived monoclonal NMDAR-Ab to quantify their action over time precisely, at the nanoscale.

## Materials and methods

### Preparation of mixed neuronal culture and transfection

Mixed cultures of hippocampal neurons and glia were prepared from Day 18 embryonic Sprague–Dawley rats, as previously described.^26^ Briefly, hippocampi were dissected and mechanically dissociated after 15 min treatment at 37°C with 0.05% trypsin–EDTA 1× (Thermo Fisher Scientific, #25300-054) solution containing penicillin–streptomycin (Thermo Fisher Scientific, #15140-122) and HEPES (Thermo Fisher Scientific, #15630-056). Cells were plated at a density of 275 000 cells per dish onto poly-L-lysine (1 mg/ml, Sigma-Aldrich, #P2636) precoated glass coverslips in 60 mm Petri dishes in Neurobasal™ Plus Medium (Thermo Fisher Scientific, #A3582901) supplemented with 0.5 mM GlutaMAX (Thermo Fisher Scientific, #35050-038), 1× B-27™ Plus Supplement (Thermo Fisher Scientific, #A3653401) and 1.5% horse serum (Thermo Fisher Scientific, #26050088). Cultures were kept at 37°C in air supplemented with 5% CO_2_. Neurons were transfected at 10 days *in vitro* using the calcium-phosphate coprecipitation method. Precipitates containing plasmid DNA were prepared using the following solutions: TE (1 mM Tris–HCl pH 7.3, 1 mM EDTA), CaCl_2_ (2.5 M CaCl_2_ in 10 mM HEPES, pH 7.2), HEPES-buffered saline (HEBS; 12 mM dextrose, 50 mM HEPES, 10 mM KCl, 280 mM NaCl and 1.5 mM Na_2_HPO_4_.2H_2_O, pH 7.2). Coverslips containing neurons were moved to 12-well multi-well plates containing 200 ml per well of conditioned culture medium. Then, 50 ml of precipitate solution was added to each well, in the presence of 2 mM kynurenic acid (Sigma-Aldrich, #K3375) and incubated for 1 h at 37°C. Afterwards, cells were washed with unsupplemented Neurobasal™ plus medium containing 2 mM kynurenic acid and moved back to their original culture dish for 4 days of expression before use. The medium was changed during transfection to BrainPhys^TM^ medium without Phenol Red (Stemcell, #05791) supplemented with 1× B-27™ plus supplement.

### Plasmid DNA

GluN2A-SEP, GluN1-SEP and GluN1-mEos3.2 were expressed in a pRcCMV plasmid with SEP and mEeos3.2 at the N-terminal. Homer1c-dsRed and Homer1c-GFP were expressed in pcDNA3.1 with dsRed and GFP at the N-terminal of the insert. Flag-EphB2R Y504E and Flag-EphB2R Y504 mutants were generated from Flag-EphB2R wild-type (WT) in a pcDNA3 plasmid, as previously described.^18^ The sh_Control and sh_EphB2R were described previously.^27^ GluN1-V5-HRP was generated in a pcDNA3 vector with V5 tag and horseradish peroxidase (HRP) at the N-terminal. Clathrin Light Chain-mCherry at the N-terminal was described previously.^28^

### Patients’ monoclonal antibodies and CSF

Monoclonal human IgG1 reacting to the GluN1 subunit of the NMDAR (NMDAR-Ab, clones #003-102, #008-218, #197-073 and #007-124) was obtained and cloned from antibody-secreting cells derived from the CSF of patients with NMDAR encephalitis.^29,30^ Expression vectors encoding for heavy and light chains of this clone were introduced into HEK293 cells through transient transfection. The supernatant was used to purify the recombinant autoantibodies, following previously established methods.^29^

The human control antibody (Control-Ab) was an antibody non-reactive to human tissue derived from mature B cells from the blood of a healthy donor.^31^

A CSF sample from an 11-year-old female patient with classic acute symptoms of limbic encephalitis (speech and memory disorders and psychiatric disturbances with seizures) and negative global tumour screening was also used in the study. The patient underwent pretreatment lumbar puncture at Bordeaux University Hospital (France). The CSF was tested for the presence of NMDAR-Ab using a cell-based assay on human embryonic kidney cells (HEK293) expressing both GluN1 and GluN2B subunits of the NMDAR, as previously described.^7^ Cells were fixed [4% paraformaldehyde (PFA), 10 min], then incubated with the patient’s CSF (1:50 in saturation buffer, 90 min). The CSF was considered as positive when a clear staining was confirmed by three different readers in three independent assays.

CSF samples were rapidly stored at −80°C after acquisition in the Multi-thematic Biological Resource Centre (Biobank of the Bordeaux University Hospital, France, certification NF S 96-900). The activity of conservation of biological material was declared under no. DC-2020-3863, and the activity of transfer of biological material was authorized by the Ministry of Research under AC-2019-3595. The subject and her legal guardian provided their written informed consent, and with the authorization of the local ethics committee of Bordeaux University Hospital.

### Live imaging

Neurons were incubated with Control-Ab, NMDAR-Ab or GFP-Ab (Thermo Fisher Scientific, #A6455) in a wet chamber at 37°C, with the indicated time and concentration. Coverslips containing neurons were then mounted in an open chamber (Ludin chamber, Life Imaging Services), with 500 µl of Tyrode solution (30 mM D-glucose, 120 mM NaCl, 5 mM KCl, 2 mM MgCl_2_, 2 mM CaCl_2_ and 25 mM HEPES, pH 7.3–7.4). For time-lapse imaging, NMDAR-Ab were added directly into the chamber. Microscope sessions were performed with an inverted confocal spinning-disk Leica DMI8 microscope equipped with 63× oil objective, a sCMOS Prime 95B camera (Photometrics) and an environmental chamber to control the temperature and CO_2_ (37°C, 5% CO_2_). A 488 nm laser was used for GluN2A-SEP and GluN1-SEP and a 561 nm laser for Homer-dsRed and CLC-mCherry. The laser power was kept the same between all conditions. The different analyses were carried out in ImageJ with a homemade macro. The density of GluN1-SEP and Homer clusters was calculated using the mean of cluster density from two regions of interest per neuron.

### Immunocytochemistry

For live surface staining, neurons were incubated for 30 min with NMDAR-Ab (1 µg/ml), EphB2R-Ab (1/200, R&D system, #AF467), anti-Flag for Flag-EphB2R WT or Flag-EphB2R mutants (1/1000, Sigma-Aldrich, #F2555) or NMDAR-Fab fragment at 37°C. Cells were fixed for 15 min in 4% PFA (Sigma-Aldrich, #P6148) and 4% sucrose (Sigma-Aldrich, #0389) in PBS, then washed several times with PBS supplemented with 50 mM NH_4_Cl (Sigma-Aldrich, #A4514). Neurons were fixed with a blocking solution containing PBS and 3% bovine serum albumin (BSA; Sigma-Aldrich, #A3059) for 1 h at room temperature (RT) and incubated for 1 h at RT with secondary antibodies (1/500, Alexa 647 goat anti-human #A21445, Alexa 488 donkey anti-goat #A11055, Alexa 488 goat anti-rabbit #A11008, Thermo Fisher Scientific) in blocking solution. For intracellular staining, cells were fixed in 4% PFA–4% sucrose, blocked, permeabilized with PBS–0.1% Triton X-100 (Sigma-Aldrich, #T9284) and incubated with MAP2 antibody (1/5000, Abcam, #ab5392) for 1 h at RT or with Homer antibody (1/400, Synaptic Systems, #160004) and gephyrin antibody (1/400, Synaptic Systems, #147111) overnight at 4°C in blocking solution. Neurons were then incubated with secondary antibody (1/500, Alexa 488 goat anti-chicken #A11039, Alexa 568 goat anti-guinea pig #A11075, Alexa 488 goat anti-mouse #A11001, Thermo Fisher Scientific) in blocking solution for 1 h at RT. Coverslips were mounted with Fluoromount (Invitrogen, #00-4958-02) and kept at 4°C until imaging.

### Coupling of latex beads with antibodies and evaluation of the efficiency of the coupling

Four micrograms of CML latex beads (1.0 µm; Invitrogen, #C37483) were centrifuged at 15 000*g* for 10 min and washed in 1 ml MES coupling buffer [MES 50 mM pH 6; EDTA 1 mM; 0.0005% Tween 20 (Sigma-Aldrich, #P9416)]. Beads were resuspended in 300 µl MES coupling buffer, and carboxyl groups were activated by adding 120 µl of freshly prepared 50 mg/ml EDAC (#E6383, Sigma-Aldrich)–MES buffer. Beads were mixed on a rotating wheel for 15 min. Activated beads were washed three times in PBS–0.0005% Tween 20 after each centrifugation at 15 000*g* for 10 min. Activated beads were resuspended in 50 µl PBS, and monoclonal antibodies were added at 1 mg/ml [Control-Ab, NMDAR-Ab, GluA2 (Merck Millipore, #MAB397)] and mixed in a thermomixer (1000 rpm, 4 h). Beads coupled with antibody were washed in PBS–0.0005% Tween 20, then resuspended in PBS–BSA 1% and kept at 4°C. To evaluate the efficiency of the coupling, beads were mixed with secondary antibody Alexa 488 (1/500, anti-mouse #A11001, anti-human #A11013) for 15 min on a rotating wheel. Flow cytometry was performed at the TBM Core platform (Bordeaux). Beads coupled to antibody were applied to the neuron at a concentration of 40 µg/ml.

### Fab fragment preparation

Fab fragments from monoclonal NMDAR IgG (clone #003-102) were prepared following the manufacturer’s instructions (Pierce™ Fab Micro Preparation Kit, Thermo Fisher Scientific, #44685). Briefly, IgG was digested for 3–4 h with a digestion buffer in a column tube containing immobilized papain, with a tabletop rocker at 37°C. Fab fragments were then purified from non-digested IgG and Fc fragments with a Protein A column. Digestion and purification were confirmed with SDS-PAGE, and the Fab concentration was determined with absorption at 280 nm.

### Single quantum dot tracking

Neurons were initially incubated for 2 h in medium from the cells containing 1 µg/ml anti-EphB2R antibody (R&D system, #AF467) in a wet chamber or directly incubated for 10 min with rabbit anti-GFP antibody (1/25 000, Thermo Fisher Scientific, #A6455) followed by 10 min incubation with QD655 coupled to goat anti-rabbit F(ab′)2 (1/25 000, Thermo Fisher Scientific, #Q11422MP). All incubations were done in Tyrode solution supplemented with 1% BSA at 37°C. Coverslips were mounted in Tyrode solution in a heated chamber for observation. Quantum dots (QDs) were detected by using a mercury lamp and appropriate excitation/emission filters. Images were obtained with an acquisition time of 50 ms (20 Hz) with 500 frames. Signals were detected using an EMCCD camera (Evolve, Photometrics). QD recording sessions were processed with the software Metamorph. The instantaneous diffusion coefficient (*D*) was calculated for each trajectory from linear fits of the first four points of the mean square displacement (MSD) versus time function using MSD(*t*) ≤ *r*^2^ > (*t*) = 4*Dt*. To determine the distribution of single QD complexes, frame stacks were obtained, and after binarization of the synaptic signal (Homer-dsRed) the QD complexes were automatically located into the synaptic or extrasynaptic compartment.

### Single particle tracking photoactivation localization microscopy

Neurons were incubated in the different conditions at 37°C. Coverslips containing neurons were imaged in an open chamber (Ludin chamber, Life Imaging Services) with 1 ml of Tyrode solution at 37°C. The chamber was mounted on a Nikon Ti Eclipse microscope (Nikon France S.A.S.) equipped with a perfect focus system, an iLas² total internal reflection fluorescence arm (Gataca Systems) an Apo total internal reflection fluorescence 100× oil-immersion objective (NA 1.49) and an ORCA-Fusion BT sCMOS camera (Hamamatsu), with a final pixel size of 65 nm. Transfected cells were detected with the Homer-GFP signal (488 nm laser), and GluN1-mEos3.2 was photoactivated using a 405 nm laser, with the resulting photoconverted single molecule fluorescence excited with a 561 nm laser. Both 405 and 561 nm lasers illuminated the sample simultaneously. To keep the number of stochastically activated molecules constant and well separated during the acquisition, the 405 nm laser power was adjusted. Acquisition was done with Metamorph software, with 2000 frames and an exposure time of 50 ms with total internal reflection fluorescence illumination to track surface GluN1 subunit-mEos. Detection and reconnection of trajectories (>10 frames) was done with the PALM Tracer plugin for Metamorph, with a Gaussian fit to determine the centroid coordinate of a single molecule. Homer-GFP was used as a synaptic marker to discriminate synaptic and extrasynaptic GluN1-NMDAR trajectories. The MSD and coefficient of diffusion were calculated as described above for single QD tracking. The confinement area was calculated as the averaged MSD at the specified time lag in the plateau (0.5–1.0 for synaptic trajectories; 1.5–2.0 for extrasynaptic ones).

### Direct stochastic optical reconstruction microscopy

After exposure to autoantibodies (Control-Ab, NMDAR-Ab or anti-GluN1 subunit antibody clone 10B11 provided by E. Gouaux), cells were incubated for 5 min with non-permeant tyramide (300 µM, Iris Biotech, #LS-4030) and hydrogen peroxide (2 µM, Sigma, #H1009) at 37°C for GluN1-HRP imaging. Neurons were fixed for 15 min in 4% PFA–4% sucrose in PBS, then washed several times with PBS supplemented with 50 mM NH_4_Cl. Cells were blocked with PBS containing 3% BSA fatty acid free (Roche, #10775835001) for 1 h at RT. Neurons were then incubated with streptavidin–Alexa 647 (1/500, Thermo Fisher Scientific, #S32357) in the blocking solution for 30 min at RT, then the neurons were washed in PBS five times and kept at 4°C until imaging. For NHS-Ester experiments, neurons were exposed to autoantibodies and then incubated 30 min at 37°C with Tyrode solution containing Alexa 647 NHS-Ester (20 µg/ml, Thermo Fisher Scientific, #A37573). Neurons were fixed with PBS–4% PFA–4% sucrose for 15 min, then washed several times with 50 µM NH_4_Cl solution and kept at 4°C until imaging. Imaging sessions were also performed on the Nikon Ti Eclipse microscope with the same equipment as described before for single particle tracking photoactivated localization microscopy (sptPALM). Lateral drift was corrected using multicoloured fluorescent microspheres (Life Technologies, #T7279 TetraSpeck). For analysis, PALM-Tracer was used to extract exact the coordinates of a localization, and SR-Tesseler was used to quantify the clustering of proteins from the localization file, as previously described.^32^ A minimum of 10 single molecule localizations and a density factor of two was used to define a cluster (based on comparison with background signals). Synaptic clusters of proteins were determined using Homer-GFP as the synaptic marker for GluN1-HRP and Homer/Gephyrin staining for NHS-Ester experiments.

### Statistical analysis

Statistical analysis was performed using GraphPad Prism v.10 (GraphPad Software, La Jolla, CA, USA). Depending on the data distribution, the Mann–Whitney U-test or Student’s two-tailed *t*-test was used to test differences between two groups, and one-way ANOVA followed by Tukey’s *post hoc* test or the Kruskal–Wallis test followed by Dunn’s *post hoc* test was used for multiple group comparisons. Two-way ANOVA followed by Tukey’s multiple comparison test was used between groups that had been split on two independent variables. All data were obtained from at least three independents experiments (cultures).

## Results

### Acute exposure to NMDAR-Ab alters only extrasynaptic NMDAR surface trafficking

**Figure 1:**
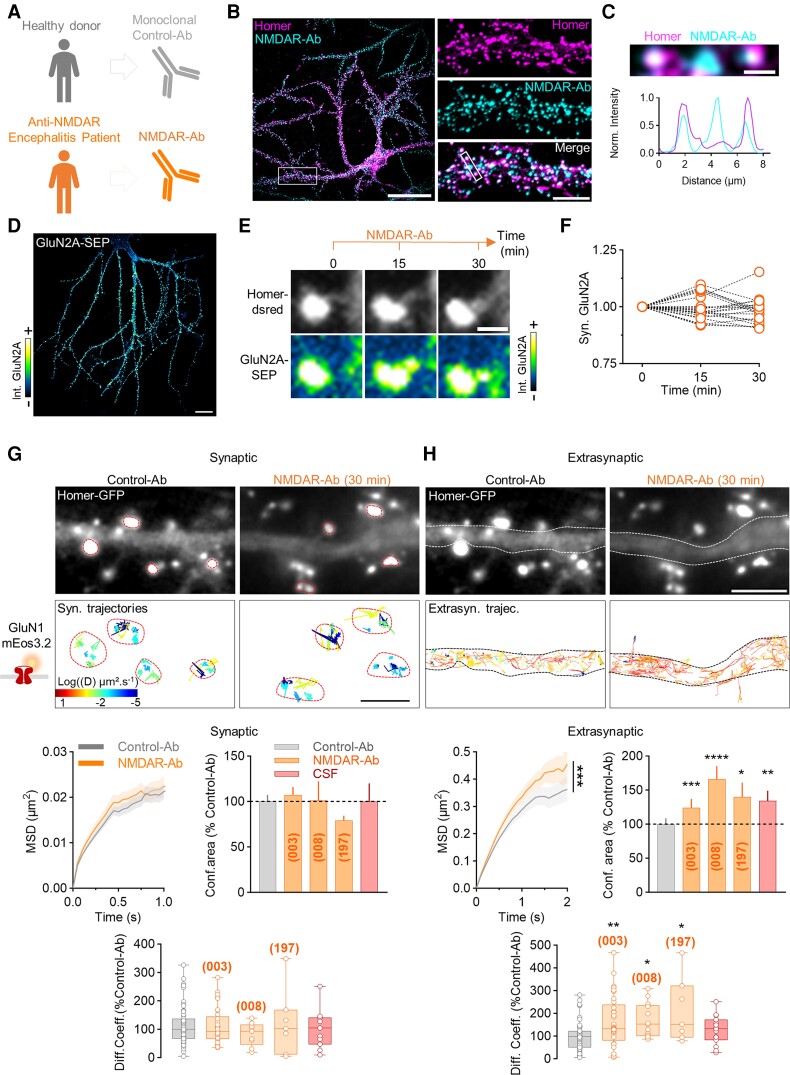
**Extrasynaptic NMDAR surface trafficking is acutely altered by NMDAR-Ab.** (**A**) Mononoclonal Control-Ab was obtained from the blood of a healthy donor, and monoclonal NMDAR-Ab was derived from the CSF of a patient with acute anti-NMDAR encephalitis. (**B**) Representative images of NMDAR-Ab (clone 003-102) surface staining (cyan) after a short incubation (1 µg/ml, 30 min) and Homer staining as a synaptic marker (magenta) on hippocampal neurons. Scale bars = 50 µm (*left*); 5 µm (*right*). (**C**) Line-scan normalized intensity of NMDAR-Ab and Homer stainings, showing that NMDAR-Ab are targeting synaptic but also extrasynaptic NMDAR clusters. Scale bar = 1 µm. (**D**) Live imaging of hippocampal neuron expressing GluN2A-SEP. Scale bar = 50 µm. (**E**) Time-lapse imaging of synaptic GluN2A-SEP during NMDAR-Ab incubation for 30 min (1 µg/ml). Scale bar = 1 µm. (**F**) Quantification of synaptic GluN2A-containing NMDAR fluorescence intensity during time-lapse imaging with NMDAR-Ab incubation. Intensity was normalized to time 0 (*N* = 5 neurons, *n* = 21 synapses). (**G** and **H**) Example neurons expressing Homer-GFP and diffusion maps of synaptic and extrasynaptic GluN1-mEos3.2. Neurons were exposed to Control-Ab, different clones of NMDAR-Ab (003-102, 008-218 or 197-073; 1 µg/ml, 30 min) or CSF from encephalitis patients. Scale bars = 2 µm (*bottom*); 5 µm (*top*). Below are shown representative mean square displacement (MSD) curves (clone 003-102), confinement area and the diffusion coefficient of synaptic and extrasynaptic GluN1-NMDAR trajectories in the different conditions. MSDs are expressed as the mean ± SEM, confinement area as the mean ± SEM and diffusion coefficient median as the median ± minimum–maximum. Each dot represents the diffusion coefficient median per neuron. Control-Ab: *N* = 55 neurons, *n* = 10 478 synaptic and 76 042 extrasynaptic trajectories; NMDAR-Ab: (003) *N* = 33, *n* = 9007 and 65 339, (008) *N* = 9, *n* = 1746 and 17 993, (197) *N* = 7, *n* = 268 and 7462; CSF: *N* = 11, *n* = 2426 and 24 633. *P < 0.05, **P < 0.01, ***P < 0.001 and ****P < 0.0001 by Kolmogorov–Smirnorv test for MSD and confinement area. *P < 0.05 and **P < 0.01 by Mann–Whitney U-test for the diffusion coefficient. Each condition was an independent experiment and was compared with the respective Control-Ab. Ab = antibody; GFP = green fluorescent protein; MSD = mean square displacement; SEM = standard error of the mean; SEP = superecliptic pHluorin.

Although the long-term effect of NMDAR-Ab on the synaptic receptor pool, transmission and plasticity has been thoroughly investigated,^10^ our understanding of the attack phase, i.e. the first minutes after exposure to NMDAR-Ab, is still limited. To tackle this question, we exposed cultured hippocampal neurons to either control monoclonal antibodies (Control-Ab) purified from the serum of a healthy donor or NMDAR monoclonal autoantibodies (NMDAR-Ab, clone 003-102) derived from NMDAR encephalitis patients (Fig. 1A). As expected, NMDAR-Ab label synaptic and extrasynaptic NMDARs following a 30 min incubation (Fig. 1B and C). To test whether NMDAR-Ab rapidly alter the NMDAR synaptic pool, we performed time-lapse imaging on hippocampal neurons expressing the GluN2A subunit tagged with a super ecliptic pH-sensitive GFP (SEP) at its extracellular N-terminus, which mainly highlight surface GluN2A-NMDARs^33^ (Fig. 1D). Over the first 30 min of exposure to NMDAR-Ab, the synaptic NMDAR fluorescence intensity was unaffected (Fig. 1E and F), indicating that the NMDAR synaptic pool was not yet affected by NMDAR-Ab in this time window. Although we previously demonstrated that NMDAR-Ab alter NMDAR trafficking in the very first hours,^7^ the methods that we used (i.e. single nanoparticle tracking) could not specifically pinpoint the locus of prime alteration. We therefore took another approach that could give us access to a high number of trajectories in each specific compartment (i.e. synaptic and extrasynaptic) simultaneously. For this, we used the super-resolution microscopy technique sptPALM, which fulfils these criteria. Hippocampal neurons were transfected with Homer1c-GFP as a synaptic marker and with GluN1 subunit coupled to the photoconvertible protein mEos3.2, which can be targeted effectively by NMDAR-Ab (Supplementary Fig. 1A). Total internal reflection fluorescence illumination was used to activate mainly surface GluN1-mEos and track synaptic and extrasynaptic NMDARs with distinct behaviours (Supplementary Fig. 1B and C). As expected, synaptic trajectories represented only ∼20% of total trajectories, and they were more confined (as indicated by the MSD curves) and less diffusive (shift towards blue colour in the diffusion map) than extrasynaptic ones (Supplementary Fig. 1C). To evaluate the acute effect of NMDAR-Ab, neurons were acutely (30 min) exposed to Control-Ab, various NMDAR-Ab clones or encephalitis patient CSF (Supplementary Fig. 1D). Synaptic NMDAR surface trafficking remained unaltered in all conditions (Fig. 1G). In contrast, the extrasynaptic NMDAR surface trafficking was strongly increased by NMDAR-Ab and patient CSF (Fig. 1H). The MSD curve was shifted left, indicating lower confinement of trajectories with a higher confinement area, and the diffusion coefficients were increased when compared with Control-Ab (Fig. 1H). Thus extrasynaptic (and not synaptic) NMDARs were similarly affected by NMDAR-Ab, irrespective of the clone. In addition, the increase in surface diffusion contrasts strikingly with the reduced diffusion produced by artificial cross-linker antibodies (Supplementary Fig. 1E). Altogether, these data demonstrate that NMDAR-Ab acutely and specifically increase the surface dynamics of extrasynaptic receptors, possibly by disruption of the protein–protein interaction between NMDAR and membrane partners.^15,16^

### EphB2R is not required for NMDAR-Ab pathogenicity

**Figure 2:**
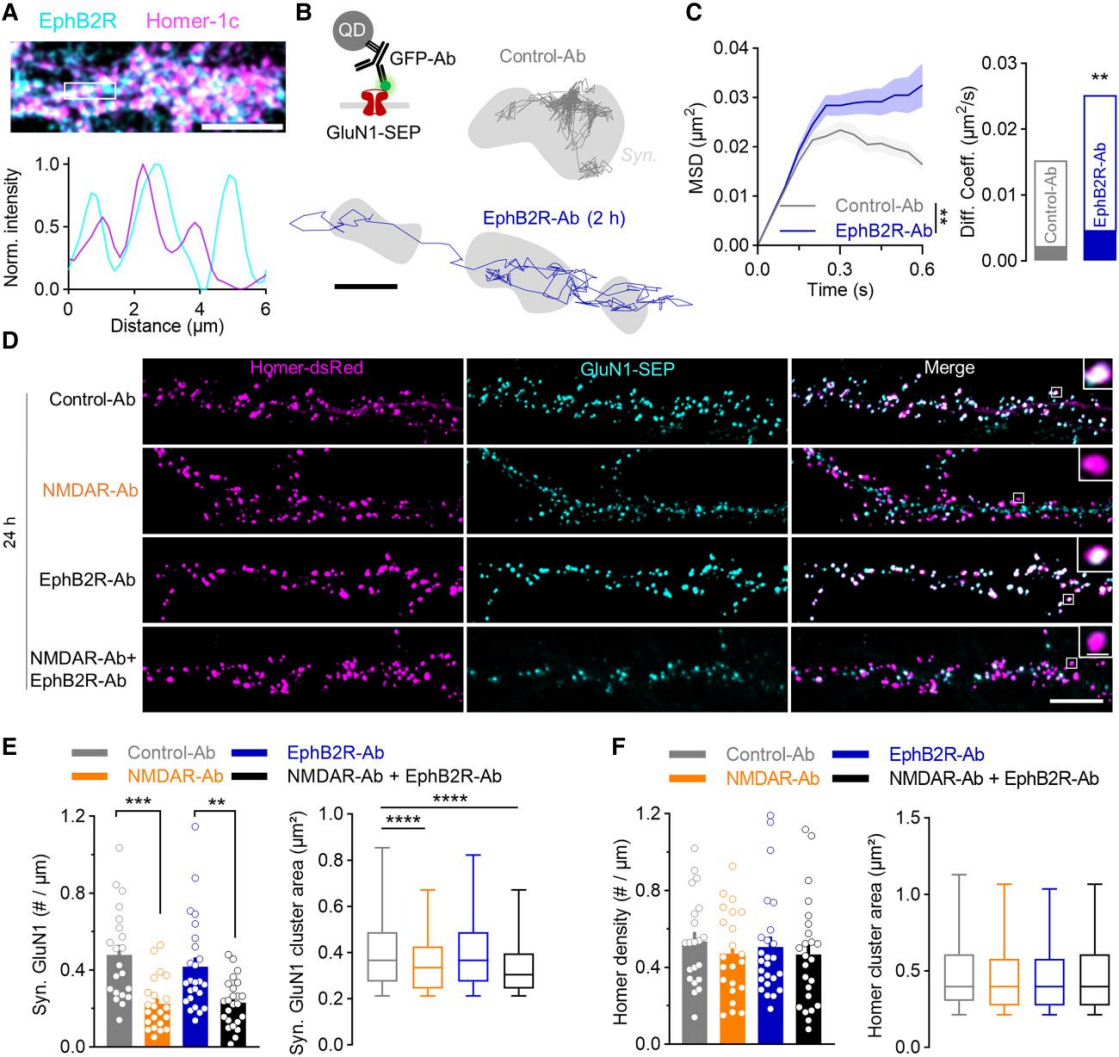
**Differential effects of NMDAR-Ab and EphB2R-Ab on GluN1-NMDAR synaptic content.** (**A**) Portion of dendrite of hippocampal neurons immunostained for Homer (magenta) and surface EphB2R (cyan). The line-scan intensity indicates that EphB2R is present at the synapse but also in the extrasynaptic compartment. Scale bar = 10 µm. (**B**) Experimental design of surface tracking of GluN1-SEP using anti-GFP antibody (GFP-Ab) and a quantum dot (QD) on neurons incubated with Control-Ab or EphB2R-Ab for 2 h (1 µg/ml). Example trajectories of GluN1-SEP at the surface of neurons incubated with the different conditions are also shown. Neurons were transfected with Homer-dsRed to delimit the glutamatergic synaptic area (in grey). Scale bar = 500 nm. (**C**) Synaptic GluN1-NMDAR mean square displacement (MSD) and diffusion coefficient of neurons treated with Control-Ab or EphB2R-Ab (2 h, 1 µg/ml). Curves of MSD are represented as the mean ± SEM, and diffusion coefficients are represented as the median ± 25%–75% interquartile range. Control-Ab: *N* = 55 neurons, *n* = 356 trajectories; EphB2R-Ab: *N* = 50, *n* = 340. **P < 0.01 by Kolmogorov–Smirnorv test for MSD and by Mann–Whitney U-test for the diffusion coefficient. (**D**) Representative live imaging of hippocampal neurons expressing Homer-dsRed (magenta) as a synaptic marker and GluN1-SEP (cyan) for surface NMDARs. Neurons were treated with Control-Ab, NMDAR-Ab, EphB2R-Ab or NMDAR-Ab + EphB2R-Ab (1 µg/ml, 24 h). Scale bars = 10 µm, 1 µm. (**E** and **F**) Quantification of synaptic GluN1-NMDAR and Homer cluster density and area of neurons treated in the different conditions. The density of clusters is represented as the mean ± SEM; **P < 0.01 and ***P < 0.001 by one-way ANOVA. Area of clusters is represented as the median ± minimum–maximum; ****P < 0.0001 by Mann–Whitney U-test. Control-Ab: *N* = 21 neurons, *n* = 1072 synaptic GluN1-NMDAR clusters and 1188 Homer clusters; NMDAR-Ab: *N* = 22, *n* = 589 and 1212; EphB2R-Ab: *N* = 25, *n* = 1140 and 1306; NMDAR-Ab + EphB2R-Ab: *N* = 24, *n* = 545 and 1128. Ab = antibody; dsRed = discosoma red fluorescent protein; GFP = green fluorescent protein; MSD = mean square displacement; SEM = standard error of the mean; SEP = superecliptic pHluorin.

Previous studies have suggested that the disruption of synaptic NMDAR–EphB2R interaction is instrumental for NMDAR-Ab pathogenicity.^7-9^ In light of the above data, we tested whether this interaction is required for NMDAR-Ab cellular effects. Initially, surface staining of EphB2R shows that EphB2R is present at the synapse and in the extrasynaptic compartment (Fig. 2A). Using an antibody directed against the extracellular part of EphB2R (EphB2R-Ab), which disrupts NMDAR–EphB2R interaction,^7^ we investigated the impact of EphB2R-Ab on NMDAR surface trafficking using single QD tracking. Neurons were incubated for 2 h with either Control-Ab or EphB2R-Ab, and single QD experiments were performed (Fig. 2B). EphB2R-Ab significantly increased synaptic and extrasynaptic NMDAR membrane dynamics (Fig. 2B and C and Supplementary Fig. 2A and B), consistent with the stabilizing role of the NMDAR–EphB2R interaction.^19^ We then tested the role of this interaction over a long antibody incubation period (24 h) by measuring, in live neurons, the synaptic content of membrane NMDARs (Fig. 2D). Compared with Control-Ab, incubation with NMDAR-Ab reduced by half the NMDAR synaptic density and decreased the cluster area (Fig. 2E). In contrast, synaptic NMDAR cluster density and area were not significantly altered by exposure to EphB2R-Ab, indicating that disrupting NMDAR–EphB2R interaction is not sufficient to deplete the NMDAR synaptic pool (Fig. 2E). Consistently, synaptic NMDAR cluster density and area in neurons exposed to both NMDAR-Ab and EphB2R were not different from NMDAR-Ab conditions alone (Fig. 2E). In all conditions, the postsynaptic density (Homer1c cluster) was not affected, indicating that the decrease of synaptic NMDAR is not attributable to the degradation of the post-synaptic density (Fig. 2F) .^5^

**Figure 3:**
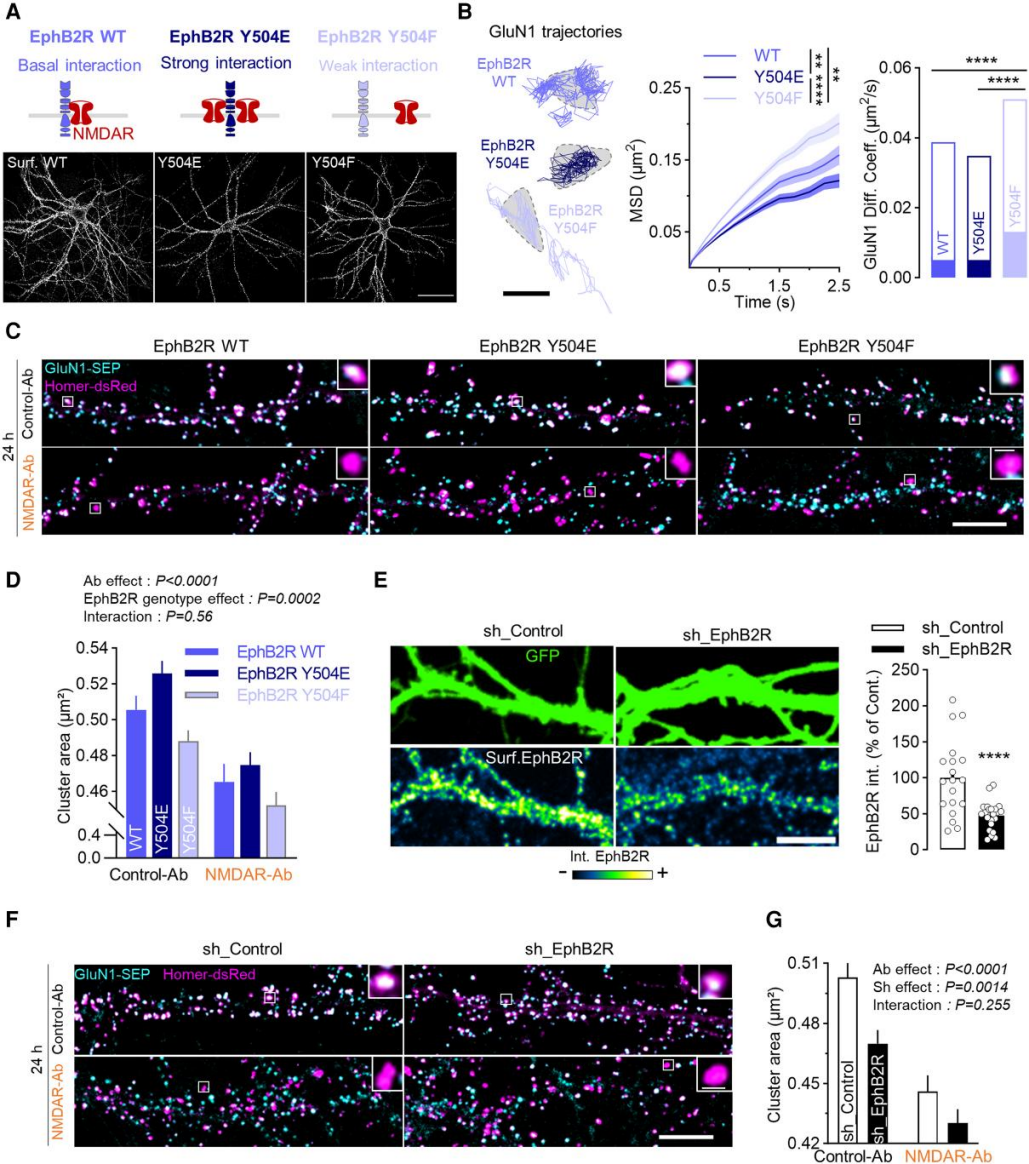
**Genetic alteration of EphB2R–NMDAR interaction impacts NMDAR dynamics but does not interfere with NMDAR-Ab.** (**A**) Schematic representation of the basal interaction between NMDAR and EphB2R (EphB2R WT), strong interaction with EphB2R-Y504F mutant and weak interaction with EphB2R-Y504F mutant. Surface immunostaining of EphB2R WT or EphB2R mutants in neurons is also shown. Scale bar = 50 µm. (**B**) Example surface GluN1-NMDAR trajectories of neurons expressing EphB2R, EphB2R Y504E or EphB2R Y504F and their associated mean square displacement (MSD) and diffusion coefficient. The MSDs are represented as the mean ± SEM; **P < 0.01 and ****P < 0.0001 by Kolmogorov–Smirnorv test. Diffusion coefficients are represented as the median ± 25%–75% interquartile range; ****P < 0.0001 by Kruskal–Wallis test. WT: *N* = 33 neurons, *n* = 1030 trajectories; Y504E: *N* = 34, *n* = 1070; Y504F: *N* = 33, *n* = 1380. Scale bar = 500 nm. (**C**) Representative live imaging of hippocampal neurons expressing GluN1-SEP, Homer-dsRed and EphB2R WT, EphB2R Y504E or EphB2R Y504F. Cells were incubated with Control-Ab or NMDAR-Ab (1 µg/ml, 24 h). Scale bars = 10 µm, 1 µm. (**D**) Quantification of synaptic GluN1-NMDAR cluster area of neurons transfected with the different EphB2R constructs and treated with Control-Ab or NMDAR-Ab (1 µg/ml, 24 h). Data are represented as the mean ± SEM; two-way ANOVA. Control-Ab/WT: *N* = 23 neurons, *n* = 1712 clusters; Control-Ab/Y504E: *N* = 31, *n* = 2421; Control-Ab/Y504F: *N* = 30, *n* = 2452; NMDAR-Ab/WT: *N* = 17, *n* = 917; NMDAR-Ab/Y504E: *N* = 27, *n* = 1450; NMDAR-Ab/Y504F: *N* = 25, *n* = 1372. (**E**) Representative images of hippocampal neurons co-transfected with soluble GFP, EphB2R-Flag and sh_Control or sh_EphB2R. Scale bar = 10 µm. On the right, quantification of surface EphB2R-Flag fluorescence intensity (mean + SEM). Each dot represents the mean intensity per neuron. ****P < 0.0001 by Student’s *t*-test. sh_Control: *n* = 19 neurons, sh_EphB2R: *n* = 22. (**F**) Representative images of live hippocampal neurons expressing Homer-dsRed and GluN1-SEP, co-transfected with sh_Control or sh_EphB2R and treated with Control-Ab or NMDAR-Ab (1 µg/ml, 24 h). Scale bars = 10 µm, 1 µm. (**G**) Quantification of synaptic GluN1-NMDAR clusters area of neurons treated with Control-Ab or NMDAR-Ab (24 h, 1 µg/ml). Data are represented as the mean ± SEM; two-way ANOVA. Control-Ab/sh_Control: *N* = 24 neurons, *n* = 1617 clusters; Control-Ab/sh_EphB2R: *N* = 27, *n* = 1719; NMDAR-Ab/sh_Control: *N* = 27, *n* = 1111; NMDAR-Ab/sh_EphB2R: *N* = 29, *n* = 1254. Ab = antibody; dsRed = discosoma red fluorescent protein; GFP = green fluorescent protein; MSD = mean square displacement; SEM = standard error of the mean; SEP = superecliptic pHluorin; sh = short hairpin; WT = wild-type.

To confirm this conclusion and strongly impair the interaction for a longer period of time, we next used a genetic strategy to modify the interaction. EphB2R–NMDAR interaction is mediated through extracellular phosphorylation of a single tyrosine of EphB2R (P*Y504).^20^ We generated two EphB2R mutants, previously characterized^18^: EphB2R-Y504E, which binds strongly to NMDAR, and EphB2R-Y504F, which binds weakly to NMDAR compared with EphB2R WT (Fig. 3A). NMDAR surface dynamics were decreased in neurons transfected with EphB2R-Y504E and increased in neurons expressing EphB2R-Y504F compared with EphB2-WT (Fig. 3B). Notably, EphB2R surface trafficking was not altered by the genetic mutations (Supplementary Fig. 3A). Using live imaging, we then tested whether NMDAR cluster density and area were altered by mutants and/or NMDAR-Ab (Fig. 3C). As expected,^17-19^ EphB2R mutants significantly altered the NMDAR and Homer1c cluster area, without affecting their linear density (Fig. 3D and Supplementary Fig. 3B). Likewise, NMDAR-Ab (24 h incubation) decreased NMDAR cluster density and area (Fig. 3D and Supplementary Fig. 3B). Interestingly, the NMDAR-Ab-induced decrease in the synaptic NMDAR pool was not altered by the EphB2R genotype (Fig. 3D and Supplementary Fig. 3B), suggesting that the action of NMDAR-Ab is independent of the state of the interaction between the NMDAR and EphB2R.

Finally, we knocked down the expression of EphB2R using a previously validated short hairpin RNA directed against EphB2R.^27^ Transfection with sh_EphB2R decreased by 50% the surface content of EphB2R when compared with sh_Control (Fig. 3E). Transfection with sh_EphB2R significantly decreased NMDAR synaptic cluster area, without a change in the linear density (Fig. 3F and G and Supplementary Fig. 3C). Incubation with NMDAR-Ab (24 h) decreased the synaptic NMDAR cluster area and cluster density (Fig. 3F and G and Supplementary Fig. 3C). Yet, the same effects were observed in the sh_EphB2R group, with no interaction between the effects of NMDAR-Ab and EphB2R knockdown (Fig. 3G and Supplementary Fig. 3C). Taken together, these results indicate that although EphB2R tunes NMDAR membrane dynamics and synaptic content, the effects of NMDAR-Ab are mainly independent from the status of the EphB2R–NMDAR interaction.

### NMDAR-Ab disorganize the NMDAR surface interactome

**Figure 4:**
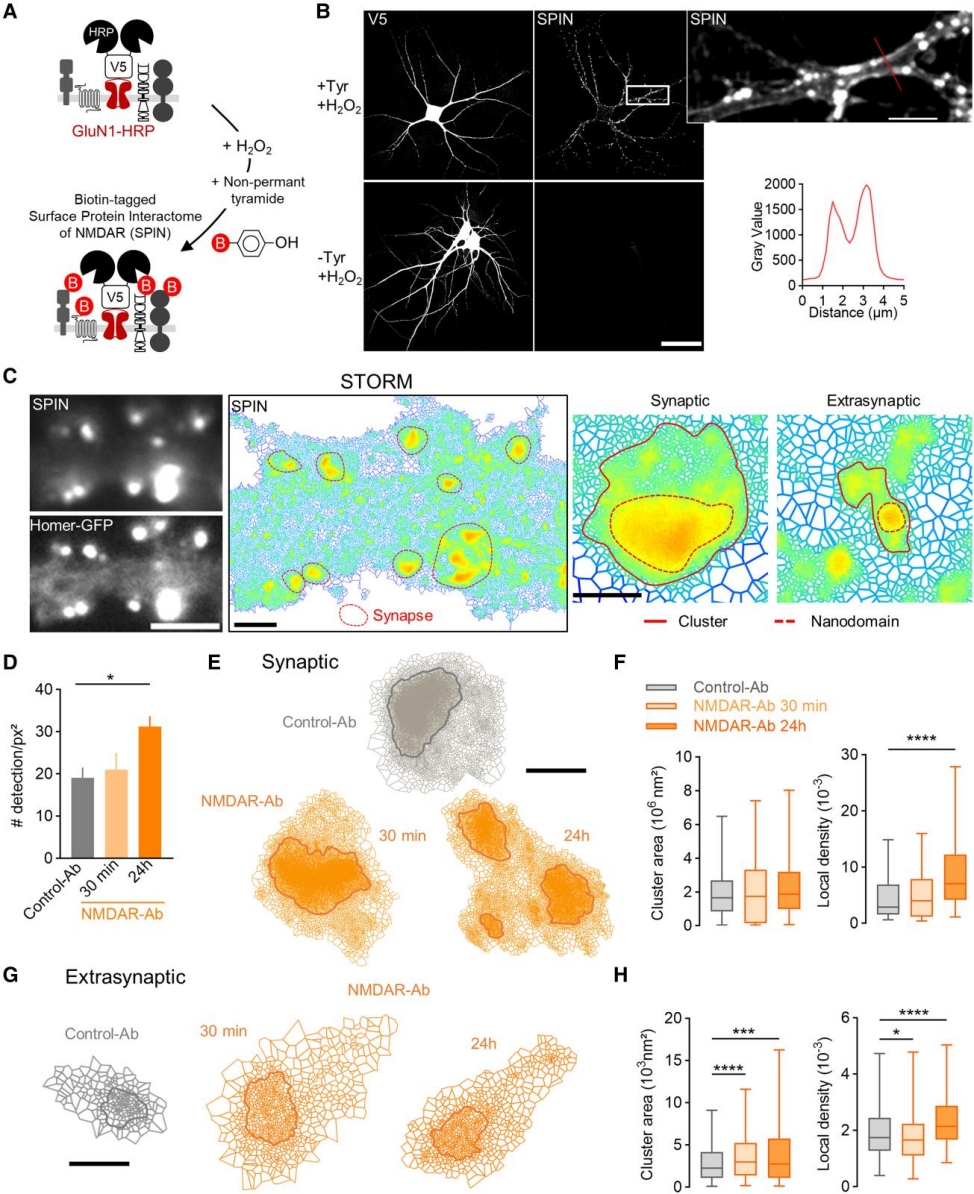
**NMDAR surface interactome nano-organization is modified by NMDAR-Ab incubation.** (**A**) Schematic diagram describing the proximity labelling assay using the GluN1-V5-HRP construct. In the presence of H_2_O_2_ (2 µM) and the biotin derivative non-permeant tyramide (300 µM) for 5 min, HRP biotinylates proteins surrounding the NMDAR within a limited radius. (**B**) Representative images of neurons expressing the GluN1-HRP construct as indicated by V5 staining. Addition of tyramide induces surface biotinylation, as shown by the line-scan intensity on the portion of dendrite. Scale bars = 40 µm, 5 µm. (**C**) The super-resolution imaging STORM reveals synaptic (within the Homer-GFP mask) and extrasynaptic SPIN nano-organization, with clusters and nanodomains obtained with SR-Tesseler. Scale bars = 3 µm, 1 µm, 250 nm. (**D**) Quantification of the number of detections per SPIN cluster area in neurons treated with Control-Ab or NMDAR-Ab (1 µg/ml, 30 min or 24 h), mean ± SEM; *P < 0.05 by Kruskal–Wallis test (Control-Ab: *n* = 21 neurons; NMDAR-Ab 30 min: *n* = 8, 24 h: *n* = 7). (**E**–**H**) Representative clusters of synaptic and extrasynaptic SPIN after incubation with Control-Ab, NMDAR-Ab (30 min) and NMDAR-Ab (24 h) obtained with SR-Tessler (scale bars = 500 nm, 100 nm), with quantification of the area of clusters and local density. Values are expressed as the median ± minimum–maximum; *P < 0.05, ***P < 0.001 and ****P < 0.0001 by Kruskal–Wallis test. Control-Ab: *N* = 21 neurons, synaptic clusters: *n* = 199, extrasynaptic clusters: *n* = 1016; NMDAR-Ab 30 min: *N* = 8, *n* = 103 and 407; 24 h: *N* = 7, *n* = 76 and 505). Ab = antibody; GFP = green fluorescent protein; HRP = horseradish peroxidase; SEM = standard error of the mean; SPIN = surface protein interactome of the NMDAR.

Whether NMDAR-Ab mediate their effect through altered NMDAR–protein interaction remains an open question. Given that dozens of membrane proteins have been shown to interact with the NMDAR,^15^ we intended to label the surface interactome of the NMDAR at the nanoscale and test whether it is impacted by NMDAR-Ab. To address this challenge, we initially generated a construct for a proximity-labelling assay, which consists of a V5-tagged GluN1 subunit coupled to an HRP enzyme (GluN1-HRP). After adding the biotin derivative non-permeant tyramide and H_2_O_2_, GluN1-HRP biotinylates only surface protein within a 20 nm radius, revealing the surface protein interactome of NMDAR (SPIN) (Fig. 4A). Neurons expressing GluN1-HRP in the presence of tyramide displayed a surface biotinylation, revealed by streptavidin, along the dendrite, with accumulation in synapses (Fig. 4B). Without tyramide, the staining was virtually absent (Fig. 4B). To characterize the nanoscale organization of SPIN within synaptic and extrasynaptic compartments, neurons were also transfected with Homer-GFP, and direct stochastic optical reconstruction microscopy imaging was performed. Clusters of synaptic SPIN were larger and denser than extrasynaptic ones (Fig. 4C). Yet, extrasynaptic SPIN clusters were numerous, paving the whole dendritic surface. Then, neurons were incubated during 30 min or 24 h with Control-Ab or NMDAR-Ab before SPIN labelling and imaging. The overall detections were increased in the SPIN after 24 h exposure to NMDAR-Ab (Fig. 4D). Acutely (30 min), NMDAR-Ab had no effect on synaptic SPIN, consistent with the above data (Fig. 4E and F and Supplementary Fig. 4A). However, NMDAR-Ab increased extrasynaptic SPIN area in both clusters and nanodomains (Fig. 4G and H and Supplementary Fig. 4B). This increase in area was accompanied by a decrease in local density. These observations are fully consistent with the increased dynamics of extrasynaptic NMDARs following NMDAR-Ab exposure, fuelling the hypothesis that NMDAR-Ab rapidly disrupt extrasynaptic complexes of NMDARs that become laterally dispersed within the membrane. In the long term (24 h), this extrasynaptic alteration fully remains and propagates to the synaptic compartment, where SPIN cluster density increased greatly (Fig. 4E–H). In addition, this chronic exposure to NMDAR-Ab altered the shape of synaptic SPIN clusters, which became fragmented into two or three nanodomains with increased local density and decreased area (Fig. 4E and Supplementary Fig. 3A). Because GluN1-Abs can be biotinylated by HRP once bound to the GluN1 subunit, we tested whether the altered SPIN nanoscale organization was attributable to the presence of antibodies. For this, neurons were acutely (30 min) exposed to an anti-GluN1 subunit antibody (clone 10B11 from rabbits), and SPIN analysis was performed. Contrary to the effect of patients’ NMDAR-Ab, this antibody reduced the area and increased the local density of extrasynaptic SPIN (Supplementary Fig. 4C), indicating that the sole presence of an antibody bound to the GluN1 subunit does not predict the effects of patients’ NMDAR-Ab. Collectively, these data indicate that a short exposure to NMDAR-Ab impairs the nanoscale organization of extrasynaptic NMDARs and related partners, with a propagation of these alterations to the synaptic receptor pool over time.

### Neuronal surfaceome is acutely altered by NMDAR-Ab

**Figure 5:**
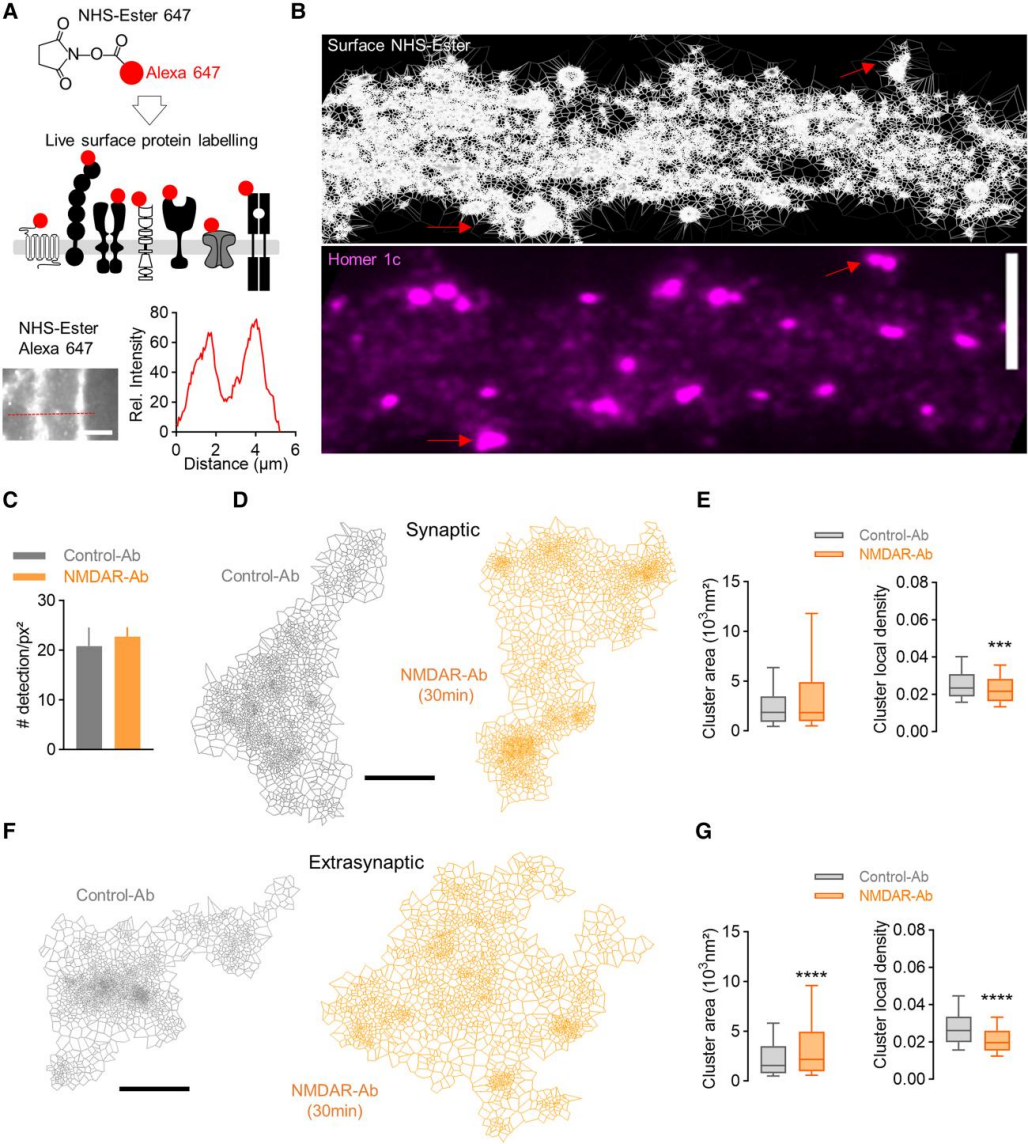
**NMDAR-Ab alter the nano-organization of all surface proteins.** (**A**) Experimental design to label all surface proteins using NHS-Ester Alexa 647. Live neurons incubated with NHS-Ester display surface staining, as shown with the line-scan intensity. Scale bar = 2 µm. (**B**) Endogenous staining of Homer and super-resolved image of NHS-Ester staining using SR-Tessler on a portion of dendrite. The red arrows indicate synapses. Scale bar = 5 µm. (**C**) Quantification of the total number of detections per surfaceome cluster area of neurons incubated for 30 min with Control-Ab or NMDAR-Ab (1 µg/ml) (mean ± SEM, *N* = 5 neurons). (**D**–**G**) Representative clusters of synaptic and extrasynaptic surface proteins after Control-Ab and NMDAR-Ab incubation (1 µg/ml, 30 min), and quantification of the area of clusters and local density. Values are expressed as the median ± minimum–maximum; ***P < 0.001 and ****P < 0.0001 by Mann–Whitney U-test. Control-Ab: *N* = 5 neurons, synaptic: *n* = 276 clusters and 244 nanodomains, extrasynaptic: *n* = 597 and 638; NMDAR-Ab: *N* = 5, synaptic: *n* = 123 and 144, extrasynaptic: *n* = 335 and 836). Scale bars = 50 nm. Ab = antibody; NHS = *N*-hydroxysuccinimide; SEM = standard error of the mean.

The fact that membrane NMDAR disorganization propagates from the extrasynaptic pool to the synaptic pool opens the possibility that many other membrane proteins could be affected. We thus labelled all membrane proteins in live neurons using NHS-Ester 647 (Fig. 5A and Supplementary Fig. 5A). Direct stochastic optical reconstruction microscopy experiments were then performed to access the nanoscale organization of all neuronal surface proteins at the surface of hippocampal neurons (Fig. 5B and Supplementary Fig. 5A and B). In the basal conditions, we report that the protein surfaceome was comparable between synaptic and extrasynaptic clusters in terms of local density and area, indicating, to our surprise, that surface proteins are packed similarly within and outside synapses (Supplementary Fig. 5C and D). NMDAR-Ab (30 min incubation) did not change the overall detection of proteins at the neuronal surface (Fig. 5C). At synapses, NMDAR-Ab impacted protein distribution modestly, with a decrease in cluster local density (Fig. 5D and E). However, NMDAR-Ab severely altered the extrasynaptic protein surfaceome. Protein clusters enlarged, and their protein density reduced massively (Fig. 5F and G). A similar effect, although to a lesser extent, was confirmed with another NMDAR-Ab clone (#007-124; Supplementary Fig. 5E and F). Together, these data indicate that a short exposure to NMDAR-Ab is sufficient to disorganize membrane proteins strongly in the extrasynaptic compartment, with an overall declustering of proteins consistent with the above increase in diffusion of receptors.

### NMDAR-Ab do not mimic artificial cross-linking

**Figure 6:**
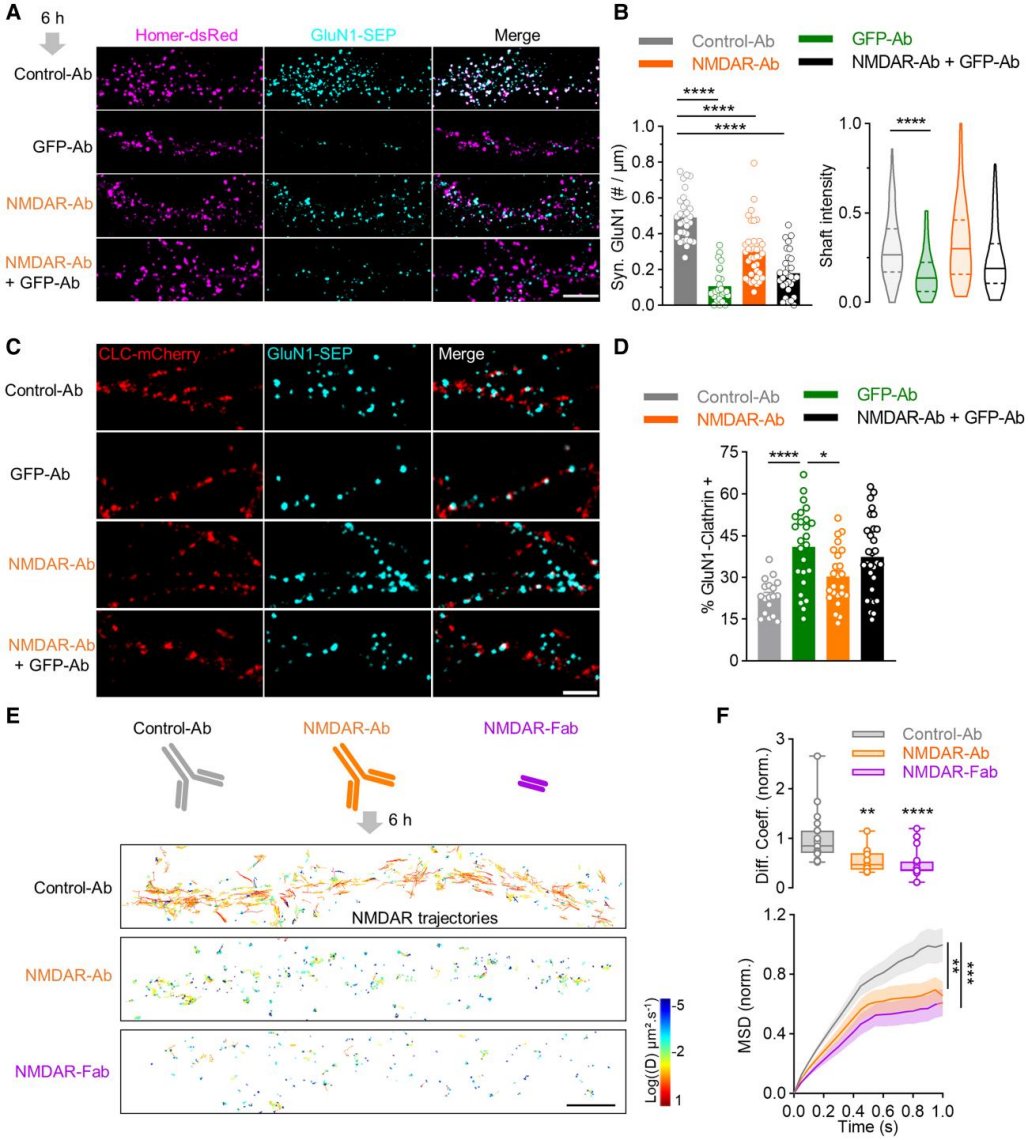
**NMDAR-Ab alters NMDAR internalization and dynamics differently from a cross-linker antibody.** (**A**) Example images of live neurons expressing Homer-dsRed and GluN1-SEP incubated for 6 h with 5 µg/ml of Control-Ab, NMDAR-Ab, GFP-Ab or NMDAR-Ab + GFP-Ab. Scale bar = 10 µm. (**B**) Quantification of synaptic GluN1-NMDAR cluster density (mean ± SEM; ****P < 0.0001 by one-way ANOVA; Control-Ab: *n* = 31 neurons; GFP-Ab: *n* = 27; NMDAR-Ab: *n* = 37; NMDAR-Ab + GFP-Ab: *n* = 30) and GluN1-NMDAR shaft intensity (****P < 0.0001 by Kruskal–Wallis test) in the different conditions. (**C**) Live imaging of neurons expressing clathrin light chain (CLC)-mCherry and GluN1-SEP in the different conditions. Scale bar = 5 µm. (**D**) Quantification of GluN1-NMDAR clusters co-localizing with CLC-mCherry (mean ± SEM; *P < 0.05 and ****P < 0.0001 by one-way ANOVA). Control-Ab: *n* = 15 neurons; GFP-Ab: *n* = 22; NMDAR-Ab: *n* = 34; NMDAR-Ab + GFP-Ab: *n* = 26. (**E**) Diffusion maps of GluN1-NMDAR after 6 h incubation with Control-Ab, NMDAR-Ab (5 µg/ml) and NMDAR-Fab (40 µg/ml) obtained with single particle tracking photoactivated localization microscopy. Scale bar = 5 µm. (**F**) GluN1-NMDAR diffusion coefficient median (median ± minimum–maximum; **P < 0.001 and ****P < 0.0001 by Kruskal–Wallis test) and mean square displacement (MSD) curves (mean ± SEM; **P < 0.01 and ***P < 0.001 by Kolmogorov–Smirnov test) both normalized to Control-Ab conditions. Control-Ab: *n* = 20 neurons; NMDAR-Ab: *n* = 13; NMDAR-Fab: *n* = 21. Ab = antibody; CLC = clathrin light chain; dsRed = discosoma red fluorescent protein; GFP = green fluorescent protein; MSD = mean square displacement; SEM = standard error of the mean; SEP = superecliptic pHluorin.

As mentioned above, NMDAR-Ab have been proposed to act as cross-linkers on the receptors, favouring their internalization. Yet, our observations of NMDAR declustering and increased lateral dynamics are simply orthogonal to such a scenario. To tackle this discrepancy, we exposed GluN1-SEP-expressing hippocampal neurons to either NMDAR-Ab or an antibody against GFP (GFP-Ab), which is one of the most potent artificial cross-linkers^34^ (Fig. 6A). GFP-Ab had no effect the SEP fluorescence *per se* or on the postsynaptic density clusters (Supplementary Fig. 6A and B). As expected, GFP-Ab (6 h) strongly reduced NMDAR cluster density in both the synapse and the dendritic shaft, indicating an overall decrease in surface NMDARs (Fig. 6B). However, the NMDAR cluster density in the dendritic shaft remained unaffected by the various NMDAR-Ab clones while the linear density of synaptic NMDAR clusters decreased (Fig. 6B and Supplementary Fig. 6C). This indicates that NMDAR-Ab (6 h) disorganize surface NMDARs without altering their membrane content. To test this possibility, neurons were exposed simultaneously to GFP-Ab and NMDAR-Ab in order to unveil a putative competitive or additive effect. NMDAR-Ab and GFP-Ab decreased the linear density of synaptic NMDARs to a similar extent, revealing no additive effect (Fig. 6B). Yet, at the extrasynaptic membrane, the exposure to both NMDAR-Ab and GFP-Ab prevented the reduction in membrane NMDAR content induced by GFP-Ab alone (Fig. 6B). Importantly, these two antibodies were not competing with each other for binding on the GluN1 subunit (NMDAR-Ab bind GluN1 subunit, whereas GFP-Ab bind SEP) (Supplementary Fig. 6D and E). Thus, the GFP-Ab-induced cross-linking of NMDARs is distinct from the effects produced by NMDAR-Ab.

To substantiate these observations, we measured the amount of NMDARs that co-localize with endocytotic pits, labelled by clathrin. For this, neurons were transfected with GluN1-SEP and clathrin light chain mCherry (CLC-mCherry) and exposed to the different antibodies for 6 h (Fig. 6C). Then, we measured the percentage of clathrin-coated pits containing NMDARs. Neurons exposed to GFP-Ab displayed a significant increase in GluN1-positive CLC-coated pits, indicating an upregulation of NMDAR internalization (Fig. 6D). In contrast, the percentage of GluN1-positive CLC-coated pits remained unaltered in neurons exposed to the NMDAR-Ab clones (Fig. 6D and Supplementary Fig. 6F). When simultaneously exposing neurons to GFP-Ab and NMDAR-Ab, the percentage of GluN1-positive CLC-coated pits remained unaltered (Fig. 6D), indicating that the NMDAR-Ab prevented the GFP-Ab-induced internalization. These data indicate that NMDAR-Ab (6 h) do not trigger a cross-linking-induced NMDAR internalization, but rather a redistribution at the neuronal surface.

The cross-linking effect of antibodies onto surface receptors relies on their divalency.^5^ To test the effect of NMDAR-Ab divalency on the receptor membrane dynamics, we generated Fab fragments from NMDAR-Ab (NMDAR-Fab) (Fig. 6E). We report that NMDAR-Fab binds to membrane receptors at high concentration (Supplementary Fig. 6G), consistent with the loss of affinity of Fab compared with full immunoglobulin (Fab_2_ + Fc).^35^ We then performed sptPALM experiments to investigate the impact of NMDAR-Ab or NMDAR-Fab (6 h incubation) on the NMDAR surface trafficking (Fig. 6E). As previously reported, neurons incubated for hours with NMDAR-Ab have reduced NMDAR membrane dynamics.^7^ Interestingly, NMDAR-Fab similarly reduced NMDAR surface trafficking, as displayed by the significant decrease in MSD and coefficient diffusion (Fig. 6E and F). Altogether, these data indicate that NMDAR-Ab rapidly disorganize extrasynaptic NMDARs and reduce, over time, their membrane dynamics in a cross-linking-independent manner.

### Targeting only extrasynaptic NMDARs with NMDAR-Ab is sufficient to induce NMDAR synaptic loss

**Figure 7:**
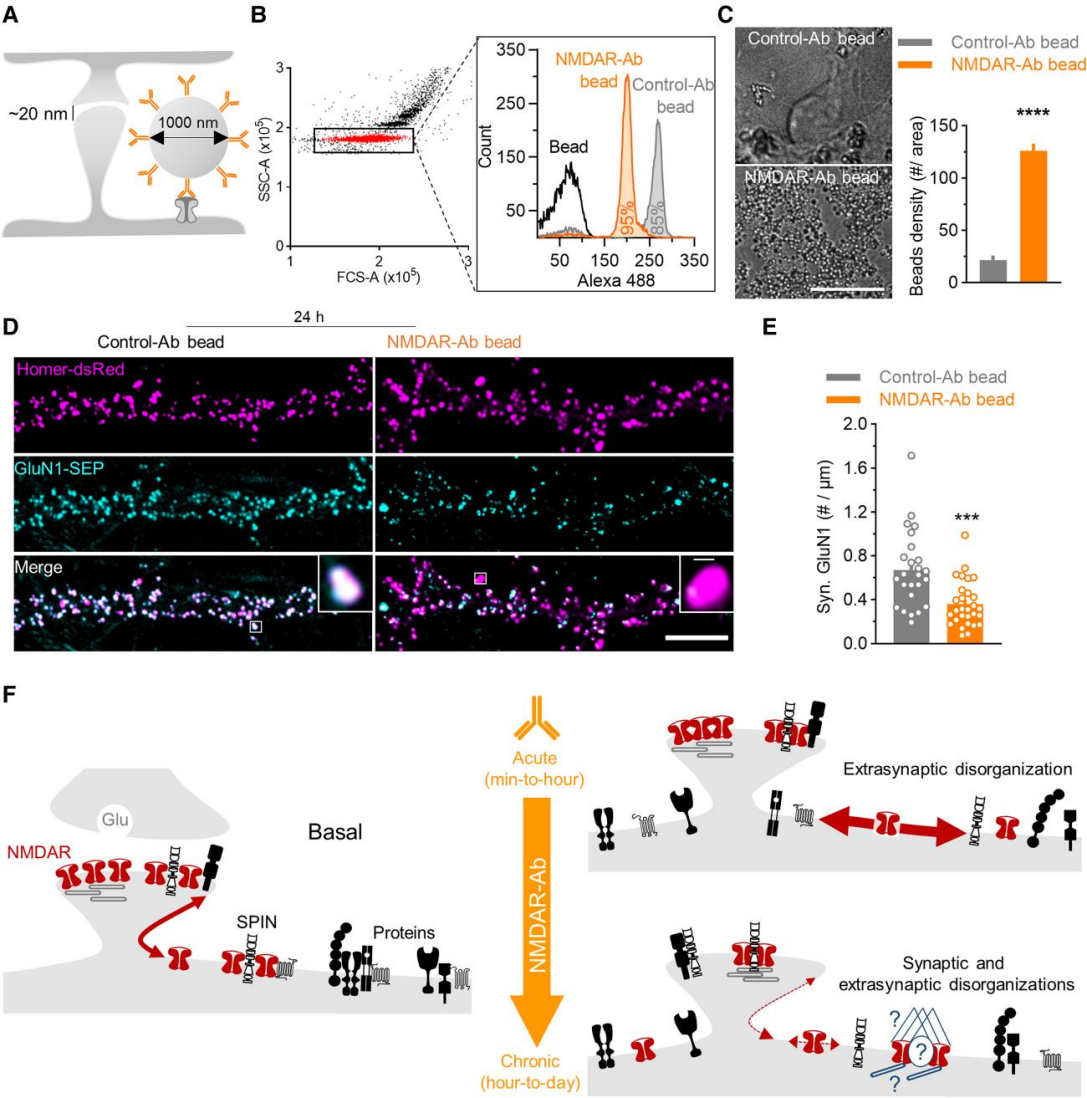
**Targeting only extrasynaptic NMDARs with NMDAR-Ab beads is sufficient to induce synaptic NMDAR loss.** (**A**) Schematic representation of NMDAR-Ab coupled to a 1 µm latex bead that can bind only to extrasynaptic NMDAR. (**B**) The flow cytometer experiment validates the efficiency of the Ab–bead coupling; in the dot plot graph, the red population corresponds to non-aggregated latex beads. From this population is extracted the fluorescence of Anti-human 488 antibody incubated with the different bead conditions: latex bead only, Control-Ab bead and NMDAR-Ab bead. NMDAR-Ab bead and Control-Ab bead curves shift to the right, meaning that the coupling was efficient, with 95% and 85%, respectively, of the total beads that were coupled effectively to the Ab. (**C**) Example images of live neurons incubated for 30 min with Control-Ab beads or NMDAR-Ab beads, with quantification of the number of beads per area (mean ± SEM; ****P < 0.0001 by Student’s *t*-test; Control-Ab bead: *n* = 8 neurons; NMDAR-Ab bead: *n* = 12). (**D**) Live imaging of neurons expressing Homer-dsRed and GluN1-SEP treated with Control-Ab bead or NMDAR-Ab bead (40 µg/ml, 24 h). Scale bar = 5 µm. (**E**) Quantification of synaptic GluN1-NMDAR cluster density (mean ± SEM; ***P < 0.001 by Student’s *t*-test; Control-Ab bead: *n* = 25 neurons; NMDAR-Ab bead: *n* = 29) of neurons treated in the different conditions. (**F**) Schematic representation of our proposed model. In basal conditions, membrane NMDARs diffuse into and out of synapses. The receptor is stabilized by intracellular and transmembrane (SPIN) proteins in both compartments. Acute exposure to NMDAR-Ab drastically disorganizes the extrasynaptic compartment: declustering of SPIN and surfaceome, and upregulating NMDAR surface trafficking. Over time, this extrasynaptic disorganization reduces NMDAR diffusion into and out of synapses, leading to a decrease in the number of synaptic NMDARs and altered synaptic nano-organization. NMDARs become sequestered in the extrasynaptic compartment through unknown mechanism(s). Ab = antibody; dsRed = discosoma red fluorescent protein; SEM = standard error of the mean; SEP = superecliptic pHluorin; SPIN = surface protein interactome of the NMDAR.

Because extrasynaptic NMDARs are the prime locus of action of NMDARs, we finally tested whether acting specifically on these receptors is sufficient to produce the pathogenic effect of NMDAR-Ab. To tackle this question, we coupled latex beads (1 μm wide) to NMDAR-Ab in order to target only extrasynaptic NMDARs (the synaptic cleft is 20 nm wide) (Fig. 7A). Using flow cytometry, we determined that 95% and 85% of the beads were successfully coupled to NMDAR-Ab and Control-Ab, respectively, without free NMDAR-Ab in the solution (Fig. 7B and Supplementary Fig. 7A). Ab-beads incubation on the neurons resulted in accumulation of NMDAR-Ab beads but not Control-Ab beads, confirming the specificity and functionality of NMDAR-Ab (Fig. 7C). To determine the effect of NMDAR-Ab beads, neurons expressing GluN1-SEP were exposed to NMDAR-Ab-beads for 24 h (Fig. 7D). Control-Ab beads had no effect on synaptic GluN1 subunit clusters or Homer1c, indicating that the treatment did not affect neuronal viability (Supplementary Fig. 7B and C). Strikingly, NMDAR-Ab beads decreased synaptic NMDAR linear density and cluster area by >50%, without altering Homer1c clusters (Fig. 7E and Supplementary Fig. 7C, D). As observed above, most of the surface NMDARs were extrasynaptic after exposure to NMDAR-Ab-beads (Fig. 7D). This effect was specific to NMDAR-Ab, because beads coupled to antibodies against the AMPA receptor subunit, GluA2, were without effect on synaptic GluN1 subunit clusters (Supplementary Fig. 7E). Together, these results indicate that NMDAR-Ab targeting only extrasynaptic NMDARs are sufficient to trigger NMDAR synaptic loss at 24 h of exposure, similar to that of NMDAR-Ab targeting both extrasynaptic and synaptic receptors.

## Discussion

Understanding the mechanism underpinning the pathogenic effect of NMDAR-Ab from patients with encephalitis is essential for the correlation with clinical symptoms, for the development of innovative therapeutic strategies for autoimmune brain disorders and for gaining further molecular insights into NMDAR-mediated neurological and psychiatric conditions. In this study, we demonstrate that various monoclonal NMDAR-Ab primarily altered the extrasynaptic NMDAR pool, and not the synaptic one. In the initial and acute phase, NMDAR-Ab greatly disorganize extrasynaptic NMDARs, membrane proteins in their close proximity and most surface proteins. NMDAR-Ab increase the dynamics of NMDARs through an overall declustering of proteins. Over time, in the chronic phase, NMDAR-Ab increase both synaptic and extrasynaptic NMDAR interactome protein density, reducing the overall membrane diffusion of NMDARs in a cross-linking-independent process. Strikingly, the full-blown effect of NMDAR-Ab was observed when they target only extrasynaptic NMDARs. Collectively, these data fuel a model in which NMDAR-Ab alter NMDAR signalling by acting initially in the extrasynaptic compartment (see model, Fig.7F). Given that the NMDAR synaptic pool depends greatly on the lateral diffusion of extrasynaptic receptors, a corrupted trafficking and organization at an extrasynaptic locus will inevitably reduce synaptic NMDARs. Our data thus support the view that NMDAR encephalitis, at its early stage, is an (extra)synaptopathy, providing a radically new perspective on the molecular mechanism and potential therapeutic perspective.

We and others have previously shown that NMDAR membrane dynamics and distribution are altered by NMDAR-Ab from patients with encephalitis and autoimmune psychosis.^7,9,36^ Using single QD tracking to determine the lateral diffusion of membrane NMDARs exposed to NMDAR-Ab, we previously showed that NMDAR dynamics were upregulated following exposure to autoantibodies.^7^ Yet, this approach allows the tracking, at a given time, of only a few NMDARs that exchange between the extrasynaptic and synaptic compartment. Here, we implemented another approach, i.e. sptPALM, because it has several key advantages for our specific question. First, sptPALM provides, at a given time, a large number of trajectories in each compartment, which contrasts strongly with single QD tracking. Second, the small size of the mEos fluorophore (3–4 nm) favours access to the synaptic cleft when compared with a QD–antibody complex (≤30 nm).^37^ Third, commercial anti-NMDAR antibodies used for single QD-NMDAR tracking might, theoretically, compete with NMDAR-Ab. Furthermore, sptPALM allows study of the impact of short-term incubation with autoantibodies, because there is no precoupling between nanoparticles and antibodies. Thanks to all these properties, it is now clearly demonstrated that the synaptic NMDAR pool is not altered by NMDAR-Ab within the first tens of minutes, or hour, contrasting with previous claims that did not have the required resolution.^7,9^

NMDAR-Ab from NMDAR encephalitis patients are classically seen as ‘cross-linkers’, based on two series of observations. First, intact immunoglobulins from an NMDAR encephalitis patient decrease synaptic NMDAR content, whereas Fab fragments from these immunoglobulins fail to do so.^5^ However, as exemplified in this study, the affinity of a Fab fragment is lower than that of a full immunoglobulin,^35^ complicating the interpretation of the data. In addition, the concentration of NMDAR-Ab and NMDAR-Fab from patients’ immunoglobulins is unknown, further limiting our capacity to draw precise conclusions. This drawback has now been circumvented by the generation of monoclonal antibodies from NMDAR encephalitis patients. Indeed, the concentration of NMDAR-Ab/Fab is now perfectly controlled and adapted to the optimal experimental setting. As expected, we had to increase the concentration of Fab fragments 10-fold to obtain a staining similar to that of full immunoglobulins, which is consistent with the loss of affinity and avidity of Fab fragments.

The second piece of evidence supporting a cross-linking effect of NMDAR-Ab arises from our past study showing that some extrasynaptic NMDARs were slowed down following hours of exposure to purified immunoglobulins from encephalitis patients.^7^ This decrease of surface dynamics resembles, to some extent, that induced by a commercial anti-NMDAR antibody.^33,38,39^ However, the surface dynamics of some extrasynaptic NMDARs was increased by immunoglobulins from encephalitis patients,^7^ which clearly contrasts with the artificial cross-linker.^33,40^ Our present study sheds new and unsuspected light. We provide direct evidence that exposure for several hours to NMDAR-Ab decreases NMDAR surface diffusion in a cross-linking-independent manner, because NMDAR-Fab produces the same effect. Future studies will be necessary to decipher the mechanism underpinning this NMDAR-Fab-induced slowing down of receptor membrane diffusion. Furthermore, whether receptors become cross-linked during the late phase of the disorder (days to 1 week) and/or whether a cross-linking process alters receptor cycling between the membrane and intracellular stores cannot be excluded. These are important issues beyond the NMDAR autoantibodies, because patient autoantibodies directed against different neurotransmitter receptors (e.g. glycine and GABA_A_ receptors) can also alter receptor-mediated ionotropic transmission, in addition to internalization through a cross-linking-independent process.^23-25,41^

NMDAR-Ab induce a massive reorganization of membrane proteins. Although this observation might be expected for NMDARs and closely related proteins, it is remarkable that the whole protein pool is affected after 30 min exposure. NMDARs represent only one protein family among >900 families identified at the plasma membrane of cultured hippocampal neurons at this developmental stage.^42^ One could thus have predicted that perturbation of the NMDAR surface trafficking and organization could go unnoticed within the whole protein surfaceome. However, we show clearly that NMDAR-Ab are sufficient to ‘declusterize’ NMDARs, their interactome (SPIN) and the protein surfaceome in the extrasynaptic compartment. We propose that the NMDAR-Ab-mediated effect relies on a broad alteration of numerous membrane proteins, as in a sequence of dominoes. Disrupting solely the interaction between the NMDAR and the EphB2R was, for instance, not sufficient to provoke the full-blown effect of NMDAR-Ab. Furthermore, a wide range of alterations of other neurotransmitter receptors and signalling cascades could be expected in such a scenario of a corrupted extrasynaptic compartment. Consistently, NMDAR-Ab strongly alter AMPA receptor- and GABA_A_ receptor-mediated transmission and synaptic pools in a process involving extrasynaptic protein–protein interactions.^43,44^ Further investigations are surely needed to disentangle the alteration of membrane protein organization in autoimmune brain disorders.

Finally, our findings demonstrate that NMDAR-Ab act independently of cross-linking in the first stage of exposure, but act as a disorganizer of the extrasynaptic compartment, in which other neurotransmitter and neuromodulator systems are strongly impaired.^43,44^ Our study points towards new therapeutic strategies in which stabilizing NMDARs at the extrasynaptic compartment might be of great interest, displacing the focus of interest from the synapse to the poorly understood extrasynaptic compartment. Other autoantibodies might also alter membrane complex interactome, providing an intriguing possibility to understand (extra)synaptopathies fully.

## Supplementary Material

awae163_Supplementary_Data

## Data Availability

Upon reasonable request, the data described in this study are available from the corresponding author.
