# Supplementary material for: NMDA receptor autoantibodies primarily impair the extrasynaptic compartment
Source: Brain. 2024 May 17;147(8):2745–60. doi: 10.1093/brain/awae163 (PMC11292910; doi:10.1093/brain/awae163)
Supplement: awae163_Supplementary_Data [file awae163_supplementary_data.pdf]

Supplementary material

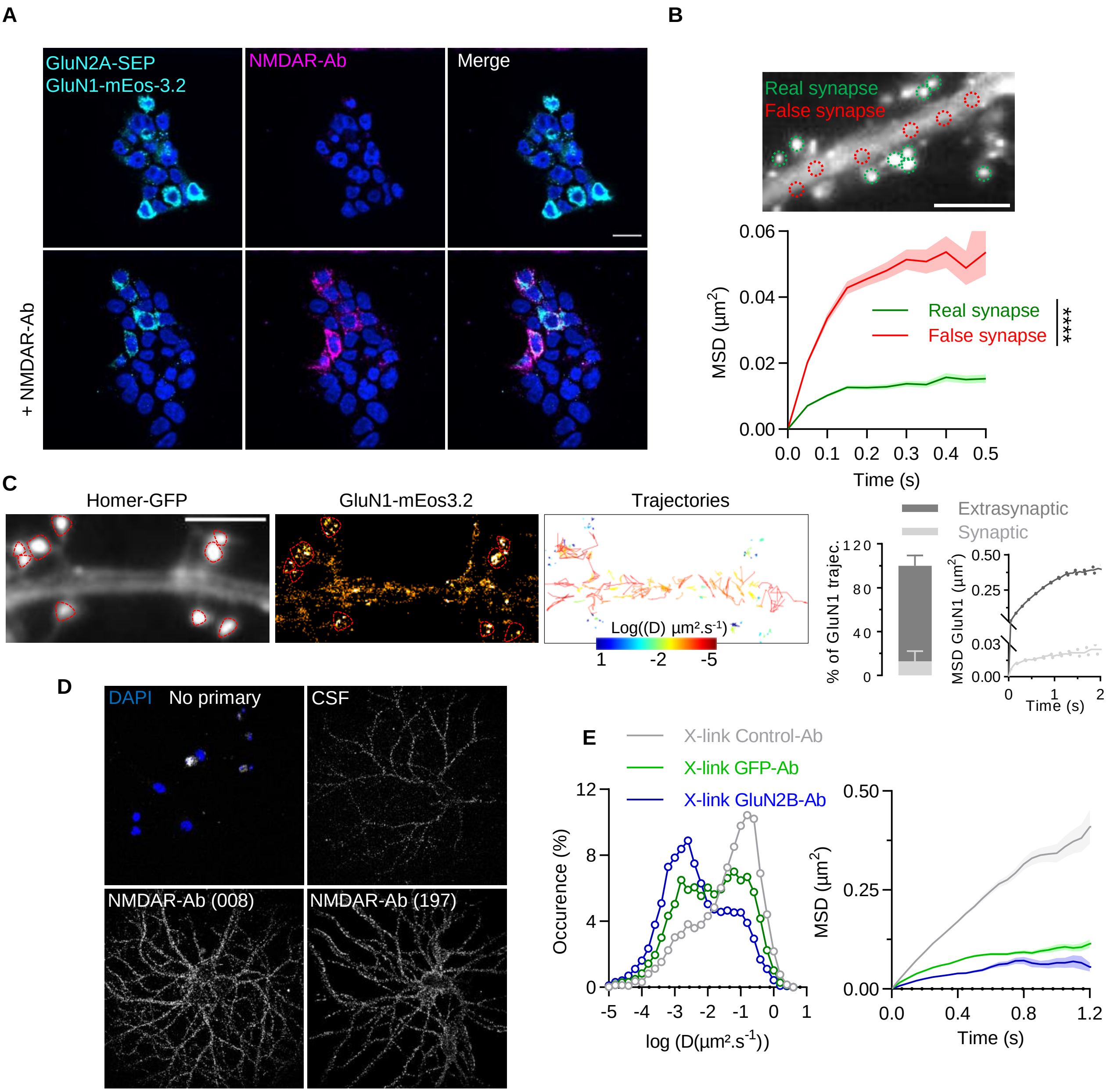

Figure S1 : Extrasynaptic NMDAR surface trafficking is acutely altered by NMDAR-Ab. (**A**) Example images of immunostaining on HEK cells transfected with GluN1-mEos/GluN2A-SEP incubated or not with NMDAR-Ab (1 µg/ml). Scale bar = 40 µm. (**B**) Image of neuron expressing Homer-GFP with ROIs defining real synapse (green) or false synapse (red). MSD of GluN1 trajectories inside the different ROI are represented and expressed as mean ± SEM (*****P*<0.0001 by Kolmogorov-Smirnov test, *N* = 5 neurons, real synapse: *n* = 3992 trajectories, false synapse: *n* = 1373). Scale bar = 5 µm. (**C**) Live imaging of neurons transfected with Homer-GFP as a synaptic marker to discriminate synaptic (red dashed circle) and extrasynaptic GluN1-mEos 3.2, scale bar = 5 µm. Using the photoconvertible properties of the recombinant receptor GluN1-mEos 3.2, sptPALM allowed to track and reconstruct GluN1 trajectories as represented on the diffusion map. Percentage of trajectories and Mean Square Displacement (MSD) curves of synaptic and extrasynaptic NMDAR are represented on the right (MSD are expressed as mean ± SEM). (**D**) Representative images of different NMDAR-Ab clones (008-218 and 197-073) and patients CSF stainings after 30 min exposure on hippocampal cultures (1µg/ml). (**E**) Diffusion coefficient distribution and MSD curves of total GluN1 trajectories of neurons incubated with Control-Ab, GFP-Ab or GluN2B-Ab in cross-linking condition (30 min, 1/10^{e}, MSD as mean ± SEM, Control-Ab : *N* = 11 neurons, *n* = 17291 trajectories; GFP-Ab : *N* = 12, *n* = 28269; GluN2B-Ab: *N* = 9, *n* = 12709).

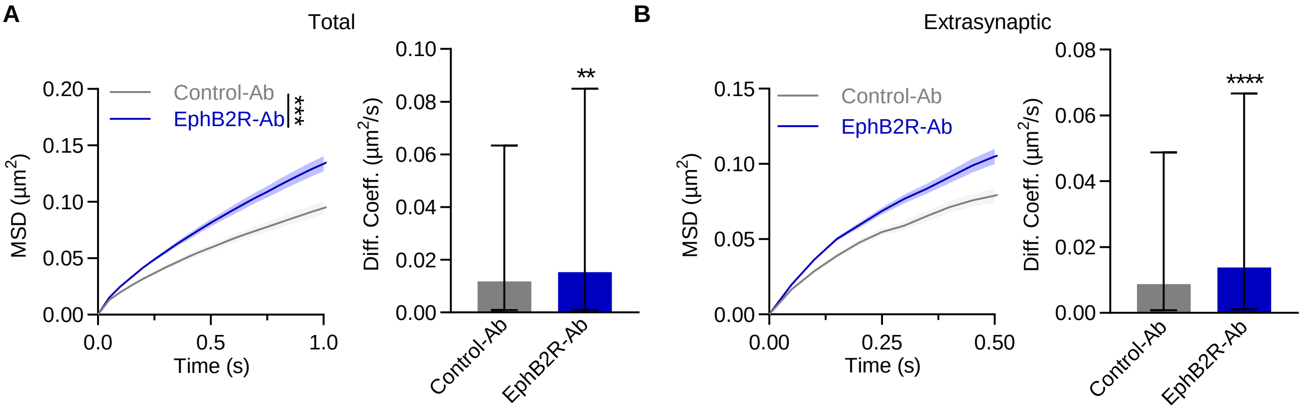

Figure S2 : Differential effects of NMDAR-Ab and EphB2R-Ab on GluN1 synaptic content. (**A-B**) MSD and diffusion coefficient of total and extrasynaptic GluN1 surface trafficking of neurons treated with Control-Ab or EphB2R-Ab (2 h, 1 µg/ml). Curves of MSD are represented as mean ± SEM and diffusion coefficient are represented as median ± IQR 25%-75% (****P*<0.001 by Kolmogorov-Smirnorv test for MSD, ***P*<0.01,*****P*<0.0001 by Mann-Whitney test for diffusion coefficient, Control-Ab : *N* = 55 neurons, *n* = 888 total and 777 extrasynaptic trajectories; EphB2R-Ab : *N* = 50, *n* = 968 and 945). (**C-D**) Quantification of total GluN1 clusters density and area after exposure to the different antibodies. Datas of cluster density are represented as Mean ± SEM, One way ANOVA test . Area are represented as median ± min to max, ****P*<0.001, *****P*<0.0001 by Kruskall-Wallis, Control-Ab : *N* = 21 neurons, *n* = 2021 clusters; NMDAR-Ab : *N* = 22, *n* = 1814; EphB2R-Ab : *N* = 25, *n* = 2362; NMDAR-Ab+EphB2R-Ab: *N* = 24, *n* = 1855).

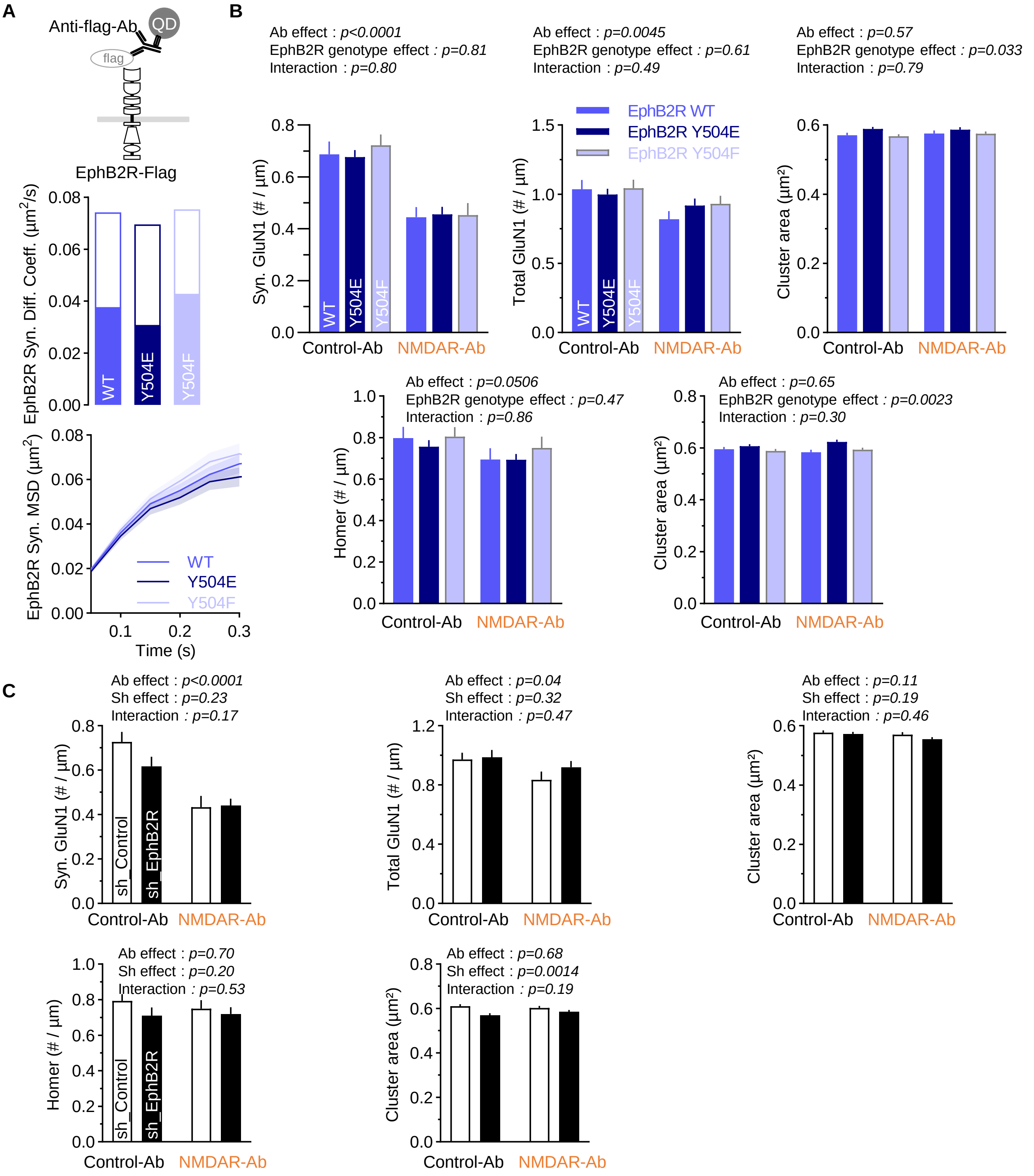

Figure S3 : Genetic alterations of EphB2R-NMDAR interaction impacts NMDAR dynamics but does not interfere with NMDAR-Ab. (**A**) Experimental design of surface tracking of EphB2R-Flag-WT or EphB2R-Flag mutants (Y504E, Y504F) using anti-Flag antibody (Flag-Ab) and a quantum dot (QD) and their associated MSD and coefficient diffusion. MSD are represented as mean ± SEM, Kolmogorov-Smirnorv and diffusion coefficient are represented as median ± IQR 25%-75%, Kruskall-Wallis (*N* = 6 neurons, WT: *n* = 317 trajectories; Y504E: *n* = 293; Y504F: *n* = 214). (**B**) Quantification of synaptic GluN1, total GluN1 and Homer clusters density (right) and area (left) of neurons transfected with the different EphB2R constructs and treated with Control-Ab or NMDAR-Ab (1 µg/ml, 24 h). Datas are represented as mean ± SEM, two-ways ANOVA test (Control-Ab/WT: *N* = 23 neurons, *n* = 1712 synaptic GluN1 clusters, *n* = 2578 GluN1 clusters, 1825 Homer clusters; /Y504E: *N* = 31, *n* = 2421, 3547, 2594; /Y504F: *N* = 30, *n* = 2452, 3619, 2713; NMDAR-Ab/WT: *N* =17, *n* = 917, 1623, 1407; /Y504E: *N* = 27, *n* = 1450, 2836, 2170; Y504F: *N* = 25, *n* = 1372, 2820, 2211). (**C**) Quantification of synaptic GluN1, total GluN1 and Homer clusters density and area of neurons transfected with the different sh_RNA and treated with Control-Ab or NMDAR-Ab (24 h, 1 µg/ml). Datas are represented as mean ± SEM, two-ways ANOVA test (Control-Ab/sh_Control: *N* = 24 neurons, *n* = 1617 synaptic GluN1, 2241 GluN1 clusters, 1800 homer clusters; /sh_EphB2R: *N* = 27, *n* = 1719, 2795, 1936; NMDAR-Ab/sh_Control: *N* = 27, *n* = 1111, 2170, 1903; /sh_EphB2R: *N* = 29, *n* = 1254, 2686, 2148).

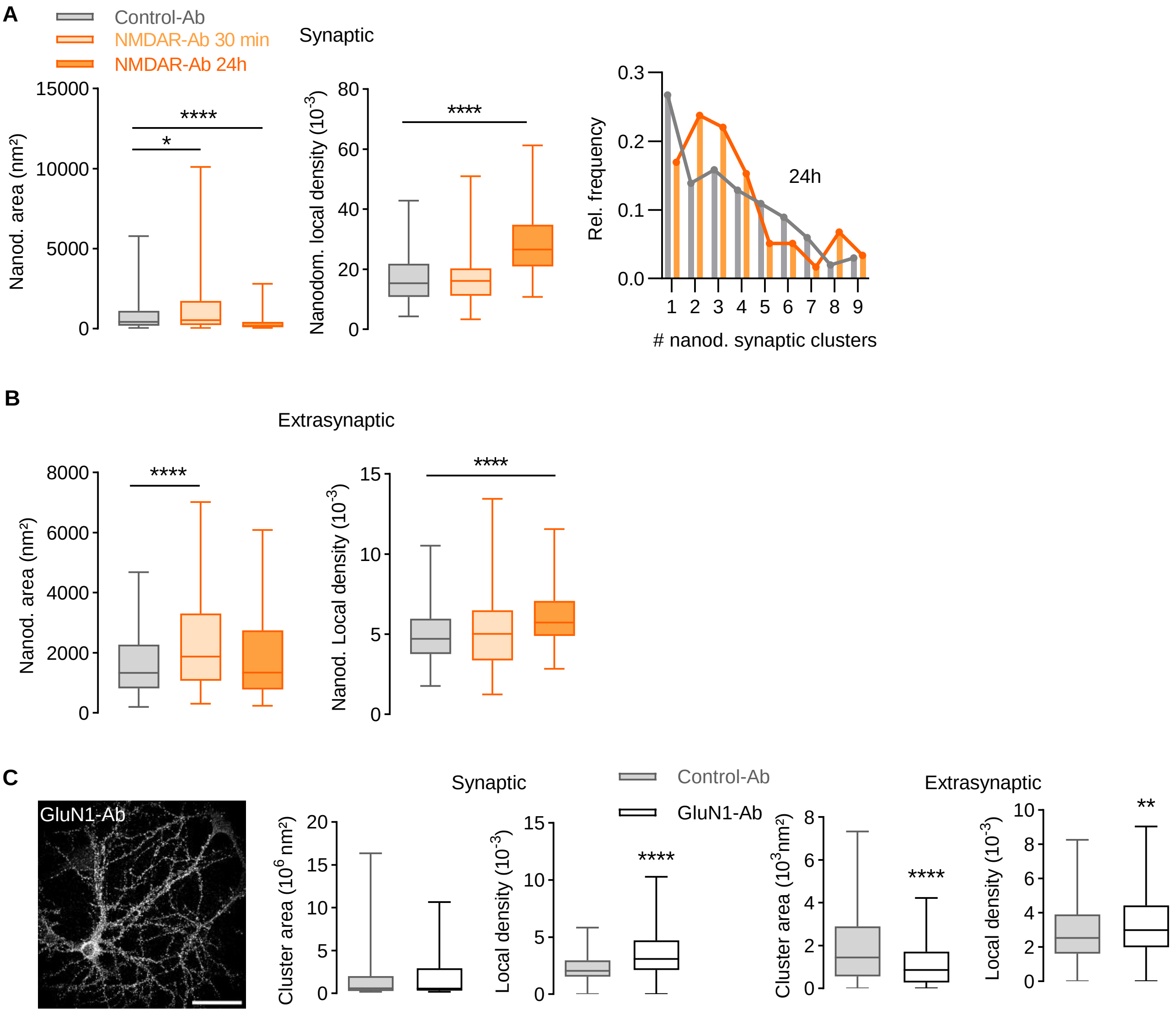

Figure S4 : NMDAR surface interactome nano-organization is modified by NMDAR-Ab incubation. (**A-B**) Quantification of synaptic and extrasynaptic SPIN nanodomains area , local density and distribution of the number of synaptic SPIN nanodomains after exposure to Control-Ab or NMDAR-Ab (median ± min to max, **P*<0.05, *****P*<0.0001 by Kruskall-Wallis, Control-Ab: *N* = 21 neurons, nanodomains synaptic: *n* = 562, extrasynaptic: *n* = 501; NMDAR-Ab 30 min: *N* = 8, *n* = 247, 259; 24 h: *N* = 7, *n* = 211, 316). (**C**) Immunostaining of monoclonal GluN1-Ab and quantification of synaptic and extrasynaptic SPIN clusters area and local density after 30 min exposure to Control-Ab or GluN1-Ab (1 µg/ml) (median ± min to max, ***P*<0.01, *****P*<0.0001 by Mann-Whitney, Control-Ab: *N* = 5 neurons, clusters synaptic: *n* = 263, extrasynaptic: *n* = 356; GluN1-Ab: *N* = 3, *n* = 96, *n* = 303. Scale bar = 50 µm.

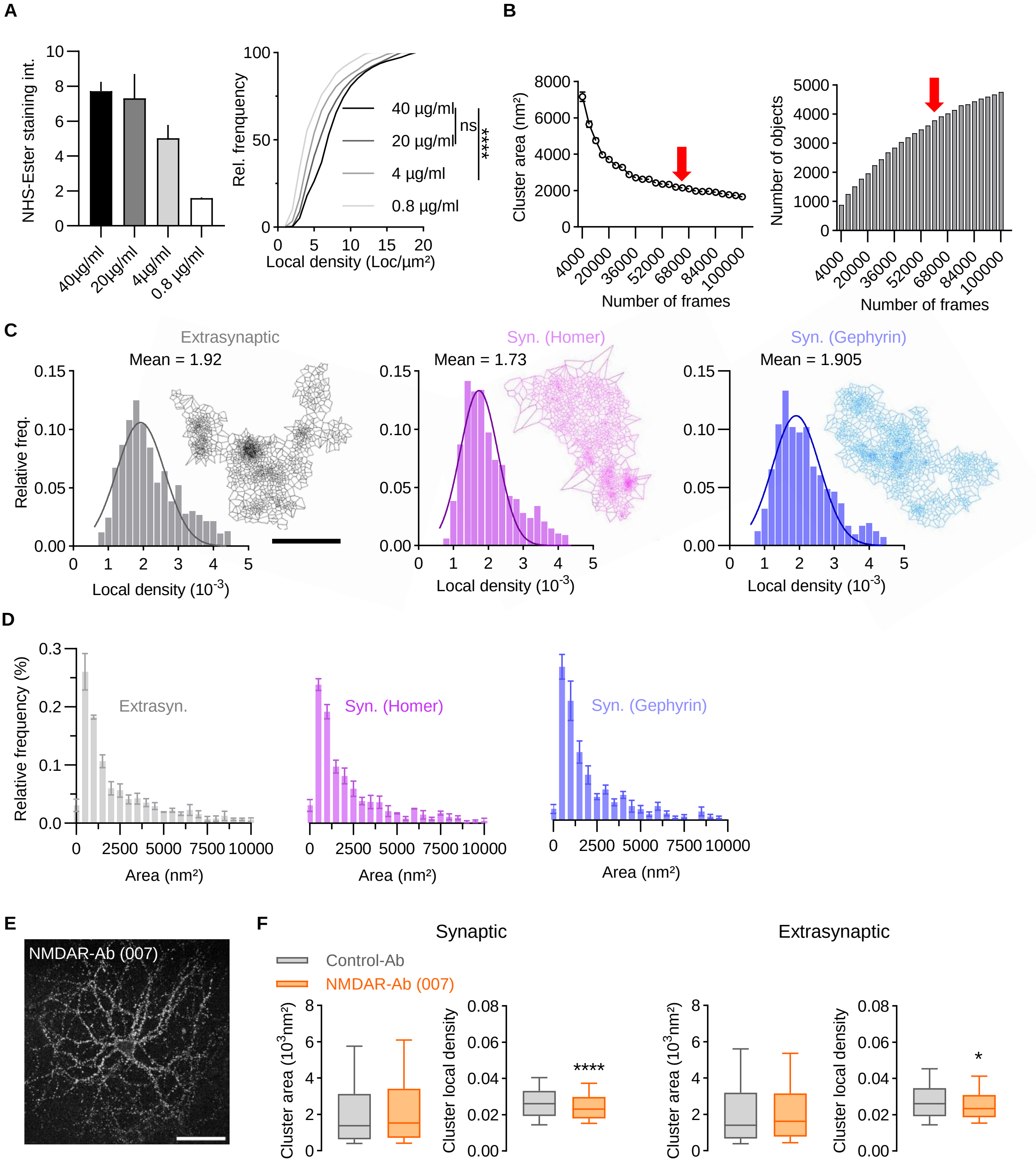

Figure S5 : NMDAR-Ab alter the nano-organization of all surface proteins. (**A**) Quantification of the fluorescence intensity (mean ± SEM) and local density (cumulative distribution) of surface NHS-Ester staining on neurons with different NHS-Ester concentration (40 µg/ml *n* = 4 neurons, 20 µg/ml *n* = 7, 4 µg/ml *n* = 8, 0.8 µg/ml *n* = 3, ****P<0.0001 by Kolmogorov-Smirnov test). (**B**) Clusters area and number of clusters detected over 100 000 frames acquisition of NHS-Ester STORM imaging. The red arrow indicates the number of frames chosen to perform analysis. (**C**) Local density distribution of synaptic and extrasynaptic surfaceome clusters labelled with NHS-Ester with their respective cluster representation. The mean of the gaussian fit is indicated. Scale bar = 30 nm. (**D**) Clusters area distribution of synaptic and extrasynaptic surfaceome clusters labelled with NHS-Ester (20 µg/ml) with their respective cluster representation. The mean of the gaussian fit is indicated. (**E**) Representative image of NMDAR-Ab (clone 007-124, 1µg/ml, 30 min) staining on neuronal culture. Scale bar = 50 µm. (**F**) Quantification of synaptic and extrasynaptic surface proteins clusters area and local density after 30 min exposure to Control-Ab or NMDAR-Ab (clone 007-124, 1µg/ml) (median ± 10-90 percentile, *P<0.05, ****P<0.0001 by Mann-Whitney, Control-Ab: N = 5 neurons, synaptic: n = 858 clusters, extrasynaptic: n = 480; NMDAR-Ab: N = 5, synaptic: n = 787, extrasynaptic: n = 366).

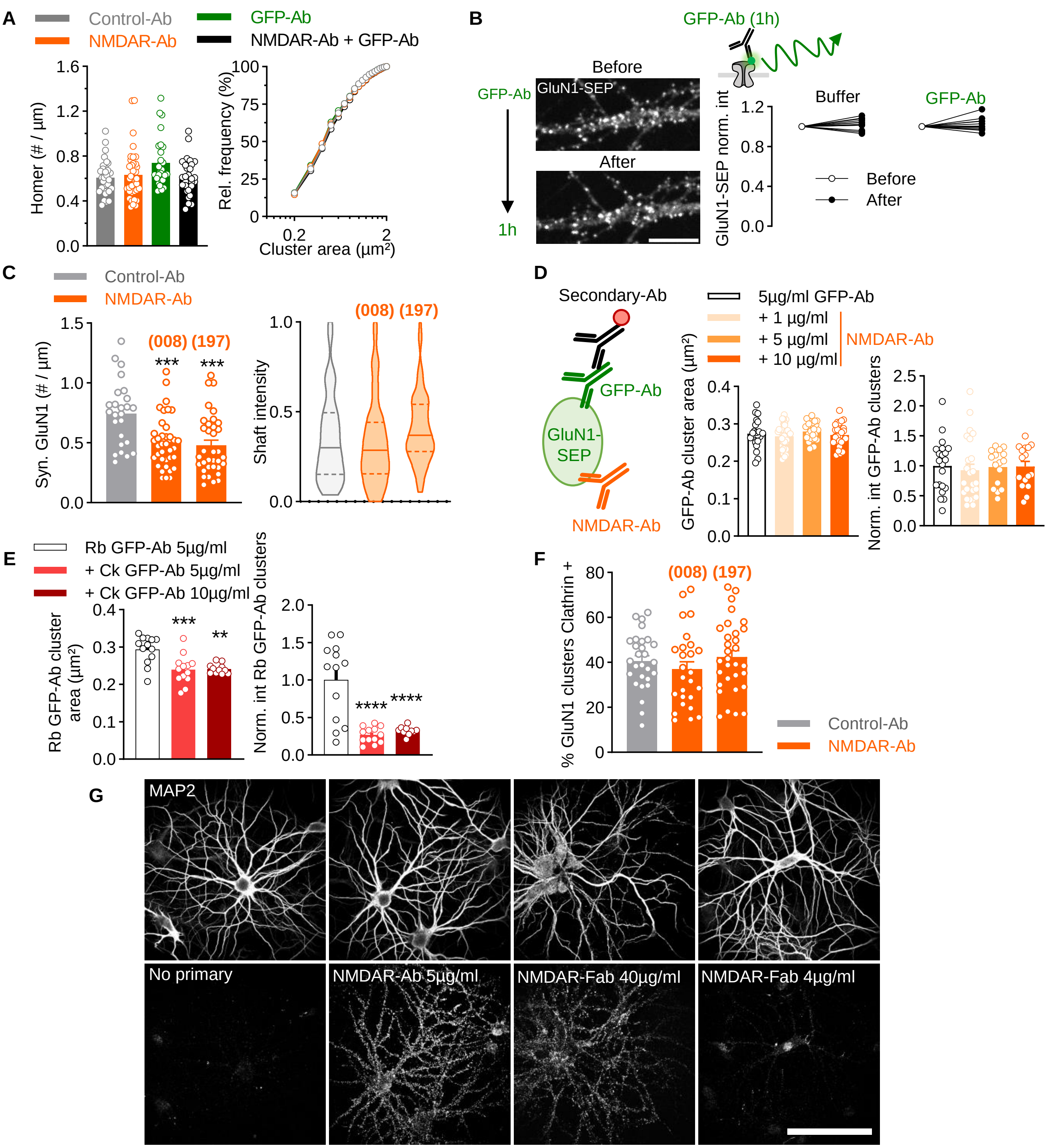

Figure S6 : NMDAR-Ab alters NMDAR organization and internalization differently from crosslinker antibody.
(**A**) Quantification of homer cluster density (mean ± SEM, one-way ANOVA) and homer cluster area (cumulative distribution, Kolmogorov-Smirnov test, Control-Ab: N = 31 neurons, n = 2365 clusters; GFP-Ab: N = 27, n = 2022; NMDAR-Ab: N =37, n =2545; NMDAR-Ab+GFP-Ab: N = 30, n = 2132). (**B**) Quantification of GluN1-SEP normalized intensity in live imaging before and after 1 h of buffer or GFP-Ab incubation (5 µg/ml). n = 6 neurons. Scale bar = 10 µm. (**C**) Quantification of synaptic GluN1-NMDAR cluster density (mean ± SEM, ***P<0.001 by ANOVA one-way, Control-Ab n = 25 neurons, NMDAR-Ab (clone 008-218) n = 37, NMDAR-Ab (clone 197-073) n = 35 and GluN1-NMDAR shaft intensity of neurons treated for 6 h with the different antibodies. (**D**) Competition assay between GFP-Ab and NMDAR-Ab for GluN1-SEP. Neurons were incubated for 30 min with 5 µg/ml of GFP-Ab alone or with different concentrations of NMDAR-Ab. Immunostaining was perform on GFP-Ab , GFP-Ab cluster area and intensity were quantified per neuron (mean ± SEM, One-way ANOVA, GFP-Ab only: n = 21 neurons; GFP-Ab+NMDAR-Ab 1 µg/ml: n = 35; 5 µg/ml: n = 27; 1 µg/ml: n = 28). (**E**) Same experiment was performed as positive control to show competition between two different GFP-Ab : Rabbit (Rb) GFP-Ab and Chicken (Ck) GFP-Ab. (mean ± SEM, **P<0.01, ***P<0.001, ****P<0.0001 by One-way ANOVA, Rb GFP-Ab only: n = 13 neurons; Rb GFP-Ab+Ck GFP-Ab 5 µg/ml: n = 11; 10 µg/ml: n = 11). (**F**) Quantification of GluN1-NMDAR clusters colocalizing with CLC-mCherry. Control-Ab: n = 28 neurons, NMDAR-Ab (clone 008-218): n = 26; NMDAR-Ab (clone 197-073): n = 31. (**G**) Representative images of neurons immunostained for MAP2, NMDAR-Ab and NMDAR-Fab. Scale bar = 50 µm.

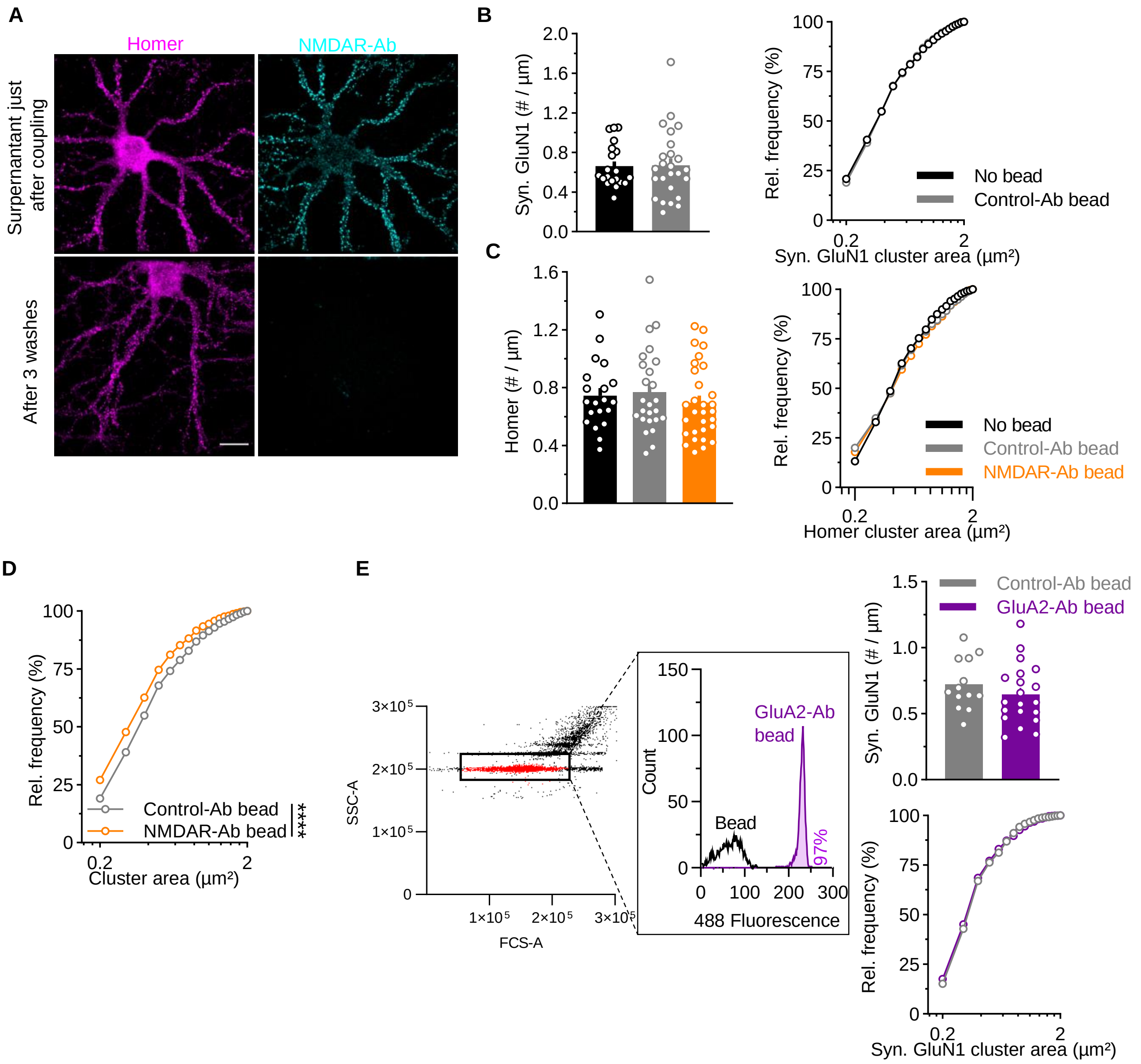

Figure S7 : Targeting only extrasynaptic NMDAR with NMDAR-Ab bead is sufficient to induce synaptic NMDAR loss. (**A**) Representative images of neurons incubated 30 min with the supernatant of the beads either just after coupling with antibodies of after 3 washes post-coupling. Neurons were stained with anti-human antibody (NMDAR-Ab in cyan) and for Homer (magenta). The supernatant contains NMDAR-Ab just after coupling but the staining is lost after 3 washes. Scale bar = 20 µm. **B-C)** Quantification of synaptic GluN1 and homer clusters density and area of neurons incubated with latex bead only, Control-Ab bead or NMDAR-Ab bead (40 µg/ml, 20 h). For cluster density, datas are represented as mean ± SEM, One way ANOVA. For aera, the cumulative distribution is represented (Kolmogorov-Smirnov, No bead *N* = 20 neurons, *n* = 2483 synaptic GluN1 clusters, 1563 homer clusters; Control-Ab bead: *N* = 25, *n* = 2262, 1133; NMDAR-Ab bead: *N* = 30, *n* = 1112 homer clusters). (**D**) Quantitication of synaptic GluN1 clusters aera (cumulative distribution, ****P<0.0001 by Mann-Whitney, Control-Ab bead: *n* = 2262 clusters, NMDAR-Ab bead: *n* = 1907) of neurons treated with the different conditions.(**E**) To test the specificity of NMDAR-Ab bead effect on GluN1-SEP clusters, beads were also coupled to GluA2 antibody with 97 % efficiency and synaptic GluN1 clusters density and area were quantified after GluA2-bead incubation (20 h, 40 µg/ml, for density mean ± SEM and Student t-test, for area cumulative distribution and Kolmogorov-Smirnov test, Control-Ab bead: *N* = 13 neurons, *n* = 807 clusters; GluA2-bead: *N* = 20, *n* = 1167).
